# Sinomenine ameliorates rheumatoid arthritis in rodent models: a systematic review and meta-analysis of anti-inflammatory and joint-protective effects

**DOI:** 10.3389/fphar.2026.1800265

**Published:** 2026-05-04

**Authors:** Ge Li, Yu-Chen Zheng, Rong He, Jin-Liang Gao

**Affiliations:** 1 College of Traditional Chinese Medicine, Changchun University of Chinese Medicine, Changchun, China; 2 Department of Rheumatology, Affiliated Hospital of Changchun University of Chinese Medicine, Changchun, China

**Keywords:** anti-inflammatory, joint-protective, meta-analysis, rheumatoid arthritis, sinomenine

## Abstract

**Objective:**

This systematic review and meta-analysis synthesize preclinical evidence to evaluate the therapeutic efficacy of sinomenine in rodent models of rheumatoid arthritis, and integrate current understanding of its potential mechanisms. This study aims to provide preclinical evidence and mechanistic insights to guide the clinical development of sinomenine for rheumatoid arthritis.

**Methods:**

Through the systematic search of 4 English and 4 Chinese databases up to November 2025, qualified studies were identified, and a total of 38 animal studies were finally included. The quality of the literature was evaluated using the bias risk assessment tool of SYRCLE, and meta-analysis was conducted using Review Manager 5.4 and Stata 18. The main evaluation indicators included clinical joint manifestations (arthritis index, paw volume), anti-inflammatory indicators (TNF-α, IL-1β, IL-6, IL-10), and joint-protective indicators (Histological score, MMP-9, RANKL, OPG).

**Results:**

The meta-analysis demonstrated that sinomenine significantly improved arthritis index and reduced paw volume. The anti-inflammatory results indicated that the levels of TNF-α, IL-1β, and IL-6 were significantly decreased, while the level of IL-10 was increased. Regarding joint protective indicators, sinomenine markedly lowered the histological score and MMP-9 levels, increased OPG levels, but showed no significant effect on RANKL levels. Subgroup analysis identified a differential dose-response, with high-dose sinomenine more consistently improving clinical and inflammatory outcomes, and the low-dose group showed relative advantages in modulating joint-protective indices. Short-term intervention demonstrated superior efficacy in reducing inflammation and providing joint protection. Intraperitoneal injection provided the most robust and reproducible efficacy profile. Sensitivity analysis showed robust results, while funnel plots revealed publication bias.

**Conclusion:**

Sinomenine alleviates systemic inflammation conditions and joint damage in rheumatoid arthritis rodents through its dual mechanisms of anti-inflammatory (inhibiting pro-inflammatory factors and promoting anti-inflammatory factors) and joint-protective (inhibiting tissue degradation and regulating bone metabolism). However, the results should be interpreted with caution due to species differences and potential publication bias. Future high-quality clinical trials are needed to verify the clinical value of sinomenine.

**Systematic Review Registration:**

https://www.crd.york.ac.uk/PROSPERO/view/CRD420251181300, identifier PROSPERO 2025 CRD420251181300.

## 1 Introduction

Rheumatoid arthritis (RA) is a chronic autoimmune disease whose main feature is symmetric polyarthritis. This leads to joint stiffness, pain, and deformity, with severe cases resulting in significant functional impairment (Guo et al., 2018; Wu et al., 2022; Ding et al., 2023; Gao et al., 2024). Over the past 3 decades, the global disease burden of RA has continued to rise, with the age-standardized prevalence and incidence increasing at an annual rate of 0.44% and 0.41%, respectively. By 2021, the total number of patients worldwide had exceeded 17.9 million, with an annual incidence of 11.80 per 100,000 population (Li et al., 2025). Importantly, RA is a systemic condition whose chronic inflammatory state significantly increases the risk of cardiovascular diseases and can lead to kidney damage (Sumida et al., 2018; Zou et al., 2017). Moreover, some extra-articular manifestations, such as those involving the lungs or mental health, can emerge even before clear joint symptoms appear (Greenblatt et al., 2020). Therefore, the prevention and management of RA remain a major challenge for global public health.

Current clinical treatment mainly relies on Western medicine, including non-steroidal anti-inflammatory drugs (NSAIDs), glucocorticosteroids (GCs), disease-modifying antirheumatic drugs (DMARDs), and biologics (Singh et al., 2025; Lin et al., 2021). Despite their wide use, these drugs have obvious limitations. A considerable number of patients have poor responses or develop resistance to them. Long-term use may also lead to adverse reactions such as gastrointestinal complications, hepatorenal toxicity, increased risk of infection, and osteoporosis, all of which seriously affect the quality of life of patients (Butola et al., 2020; Mitrović et al., 2023; Lin et al., 2020).

Due to these limitations, there is growing interest in the potential therapeutic value of botanical drugs (Chatterjee et al., 2024; Kaur et al., 2024). One such plant is *Sinomenium acutum* (Thunb.) Rehder and E. H. Wilson [Menispermaceae] (Royal Botanic GardensKew, 2026). Traditional Chinese medical texts provide detailed documentation of its use. The Ben Cao Gang Mu records its application for “wind-dampness with pain, joint swelling and immobility, numbness and itching” (Li, 1578). This traditional use is continued in modern official pharmacopoeias. Its dried caulis is documented in the Chinese Pharmacopoeia as “Qing Feng Teng” (Sinomenii Caulis) with the functions of “dispelling wind-dampness and unblocking meridians” (Chinese Pharmacopoeia Commission, 2019). In the Japanese Pharmacopoeia, the same plant is recorded as “Bōi” (Sinomeni Caulis et Rhizoma) (Ministry of Health, Labour and Welfare of Japan, 2021). These documented applications establish *S. acutum* as a well-recognized botanical remedy for arthritic conditions in East Asian traditional medicine.

The primary bioactive alkaloid derived from *S. acutum* is sinomenine (Zhou et al., 2025), which has become the focus of extensive pharmacological research. Among the numerous natural products investigated for RA, sinomenine exhibits a distinct mechanistic advantage. Unlike icariin, which mainly acts on macrophage polarization, or resveratrol, which has broad-spectrum effects on multiple cell types including T cells, osteoclasts, and chondrocytes, sinomenine preferentially targets fibroblast-like synoviocytes through inhibition of the TLR4/MyD88/NF-κB pathway (Liao et al., 2025). At the mechanistic level, sinomenine has been shown to reduce the phosphorylation of IκBα and NF-κB p65, thereby decreasing pro-inflammatory cytokine production and alleviating systemic inflammation (Lan et al., 2016). Studies on bone destruction have confirmed that sinomenine inhibits osteoclast formation and protects bone by regulating bone metabolism (Li et al., 2013). These effects complement its broader therapeutic properties, including anti-inflammatory, immunosuppressive, antioxidant, and anti-apoptotic activities (Liu et al., 2022), suggesting its potential value for various inflammatory and autoimmune diseases (Yang and Chi, 2023; Liu et al., 2016). Accumulating evidence has further elucidated the mechanisms of sinomenine in RA treatment, providing a strong pharmacological basis for its clinical application. Sinomenine-based formulations (e.g., Zhengqing Fengtongning) have been used for RA clinical treatment in China (Xiang et al., 2023).

However, existing animal studies exhibit substantial heterogeneity in key experimental parameters, including dosage, intervention duration, administration route, and outcome measures. This heterogeneity hinders a comprehensive understanding of its pharmacological effects and mechanisms (An et al., 2025; Su et al., 2025), and also results in a lack of systematic preclinical evidence to guide the optimization of dosage, duration, and administration route in clinical practice. Although recent reviews have summarized the mechanisms of sinomenine from different perspectives (Wang et al., 2025; Zong et al., 2025), these studies are qualitative in nature and have not yet quantitatively integrated key issues such as dose-response relationships and treatment regimen optimization in preclinical research. Therefore, we conducted a meta-analysis of studies using rodent models of RA to clarify whether the primary mechanism of sinomenine is anti-inflammatory and joint-protective, to examine how key experimental variables such as dose, intervention duration, and administration route influence therapeutic outcomes, and to systematically summarize the reported mechanisms of sinomenine. By providing the first integrated preclinical evidence on sinomenine in RA, this study offers a foundation for optimizing experimental protocols in future research and informing evidence-based therapeutic strategies.

The protocol was registered on the PROSPERO international register (ID: CRD420251181300) during the study design phase.

## 2 Methods and materials

### 2.1 Search strategy

We searched under the guidance of the study designer. To identify relevant animal studies investigating the effects of sinomenine on RA, we systematically searched four major English-language databases (PubMed, Cochrane Library, Web of Science, Embase) and four Chinese-language databases (CNKI, VIP, Wanfang, SinoMed). Our search scope covered all records from the inception of each database until November 2025, and the search strategy employed terms related to the subject (“sinomenine,” “cucoline”), and (“Rheumatoid Arthritis,” “Rheumatoid,” or “Arthritis”). No restrictions were applied regarding publication year, language, or blinding methods. In addition, reference lists of relevant articles were manually searched to identify any additional studies that might have been missed. All retrieved records were imported into EndNote 21 for systematic management and deduplication. Detailed search strategies for each database are provided in Supplementary File S1.

### 2.2 Inclusion criteria and outcome measures

The inclusion criteria were as follows: (1) original *in vivo* studies utilizing rodent models of RA, with no restrictions imposed on species, strain, sex, age, weight, or model type; (2) only sinomenine was used as a single intervention therapy, compared against a blank control or vehicle, and there were no restrictions on the route, time, dose and treatment regimen of administration (including preventive or therapeutic administration); (3) the treatment disease was RA; (4) studies were required to report outcomes from at least two of the following three categories: clinical joint manifestations (arthritis index and/or paw volume), anti-inflammatory indicators (TNF-α, IL-1β, IL-6 and/or IL-10), and joint-protective indicators (Histological score, MMP-9, RANKL and/or OPG). We explicitly excluded reviews, conference literature, studies involving non-rodent animals, intervention measures containing drugs other than sinomenine, and studies that failed to report the specified outcome categories above. Additionally, we excluded duplicate publications, studies with incomplete data, and articles for which the full text was unavailable. If a study involves multiple doses of sinomenine, these dose groups were analyzed as separate subgroups to ensure a one-to-one correspondence between the control group and each dose group. This study did not extract the specific detection methods for each indicator.

### 2.3 Data extraction

To ensure data accuracy and minimize individual bias, this study followed the guidelines of the Handbook of Interventions for Systematic Reviews (Version 6.5, 2024) (Higgins et al., 2024). Two independent reviewers performed the data screening and extraction. If there were differences in the extracted data, the two reviewers would first have a discussion; if the issue were not resolved, the principal investigator would make the final decision to reach a consensus. The extracted data included: (1) the baseline information included in the study, including the name of the first author and the year of publication; (2) rodent characteristics, including animal species (rat or mouse) and strains, sex ratio, age, body weight, sample size and type of animal model; (3) interventions in experimental versus control groups, route of administration and intervention time; (4) Outcome measures. Numerical values were extracted from text, tables, and figures. If the literature presented data only in the form of images, the data were extracted three times using GetData and averaged. When essential data were not reported, corresponding authors were contacted for the original values. Outcomes reported as mean ± standard error of the mean (SEM) were converted to mean ± standard deviation (SD) using the formula SD = SEM × $\sqrt{n}$ (n represents the sample size). In studies involving multiple intervention groups, only the data related to RA from the control group and sinomenine-treated groups were included.

### 2.4 Quality assessment

The potential quality risk of bias within the included primary studies was assessed using the SYRCLE assessment tool (Hooijmans et al., 2014). The assessment items covered seven fundamental bias aspects: Selection bias, Performance bias, Detection bias, Attrition bias, Reporting bias, and Other bias, totaling 10 questions. Assessments were performed within Review Manager 5.4 software, where each item was scored as having a low, unclear, or high risk of bias.

### 2.5 Statistical analysis

Meta-analysis was conducted using Review Manager 5.4 and Stata 18 software platforms. For continuous outcome measures, the pooled treatment effect was quantified using the standardized mean difference (SMD) along with its 95% confidence interval (95% CI), and forest plots were generated. The I^2^ statistic and its significance level (α = 0.1) were used to assess heterogeneity. A random-effects model was employed when significant heterogeneity was indicated (I^2^ > 50% or *P* < 0.1); otherwise, a fixed-effects model was applied. Subgroup analyses were conducted on the results with high heterogeneity to explore their potential causes. The analysis focused on evaluating the impact of different doses (divided into ≤50 mg/kg/day, 51–100 mg/kg/day, and >100 mg/kg/day), administration routes (oral gavage, subcutaneous injection, intraperitoneal injection, or intra-articular injection), and intervention duration (short-term ≤21 days and long-term >21 days) on the outcome indicators. Additionally, to assess the robustness of the pooled results and further identify studies that might introduce heterogeneity, a leave-one-out sensitivity analysis was conducted. This method involved removing each individual study sequentially and recalculating the overall effect size. We specifically observed whether the significance and direction of the pooled SMD changed, and whether the heterogeneity (I^2^) was substantially altered upon the removal of any particular study. The publication bias was evaluated by examining the funnel plot and conducting statistical tests using Egger’s regression in Stata 18. When the *P* value < 0.05, it can be considered that there was statistically significant publication bias.

## 3 Results

### 3.1 Study selection and characteristics

**FIGURE 1:**
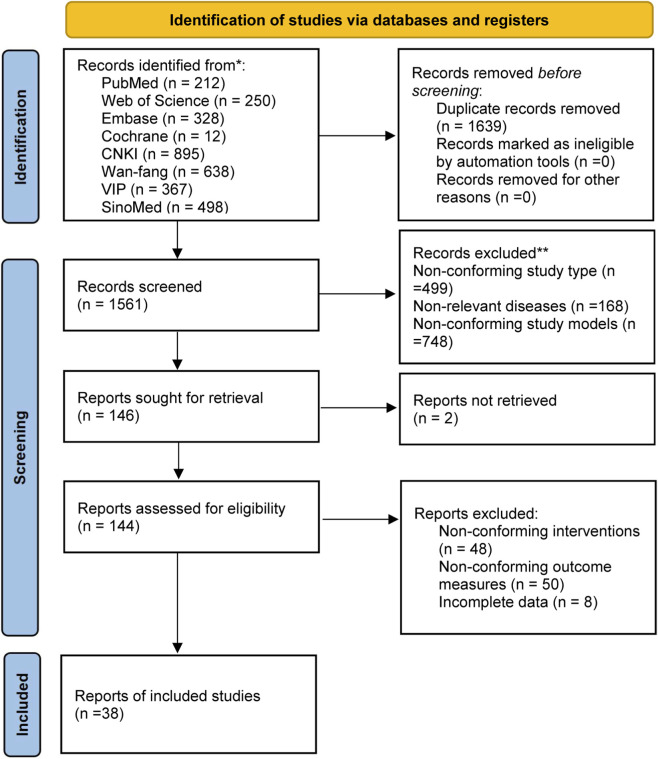
Flow diagram for systematic literature search.

As shown in (Figure 1), our search strategy identified a total of 3,200 articles. English database searches identified 802 records, of which 212 were from PubMed, 250 from Web of Science, 328 from Embase, and 12 from the Cochrane Library. Chinese database searches obtained 2,398 records, including 895 from CNKI, 638 from the Wan Fang database, 367 from the VIP database, and 498 from SinoMed. After duplicates were removed, 1,561 articles remained. Among them, 499 were reviews or other meta-analysis literature, 168 were outside the scope of our analysis, 748 were non-animal experiments, 2 were not retrieved, 48 were excluded due to incompatible interventions, 50 had non-conforming outcome measures, 8 had incomplete data, and 38 articles were finally obtained.

Table 1 summarizes the basic characteristics of the included studies. Among the 38 studies, Sprague-Dawley (SD) rats were used in 16 (42.1%), Wistar rats in 10 (26.3%), mice in 11 (28.9%), and one study (2.6%) did not specify the rat strain. Correspondingly, 355 SD rats, 318 Wistar rats, 243 mice, and 24 unspecified rats received sinomenine. The applied RA models included collagen-induced arthritis (CIA), adjuvant-induced arthritis (AIA), and antigen-induced arthritis (AA). We divided the sinomenine dose in the literature into ≤50 mg/kg/day, 51–100 mg/kg/day, and >100 mg/kg/day, and there were 22, 22, and 15 studies. The administration routes were classified as oral gavage, subcutaneous injection, intraperitoneal injection, and intra-articular injection, with 28, 1, 7, and 3 studies in each category. The intervention duration was categorized as short-term (≤21 days) and long-term (>21 days), which included 16 and 22 studies. It should be noted that some studies involved interventions with multiple doses or administration routes, and one study did not specify the route of administration. The outcome measures were primarily grouped into three categories: clinical joint manifestations (reported in 36 studies), anti-inflammatory indicators (reported in 34 studies), and joint-protective indicators (reported in 18 studies).

**TABLE 1:** Baseline characteristics of the included studies.

Number	Study ID	Sample size (sin/con)	Species	Model	Sin dose (mg/kg/day)	Administration route	Intervention duration	Outcome measures
1	Guo et al. (2025)	8/8	DBA/1 mice	CIA	100	Oral gavage	21 d	Arthritis index, histological scores, MMP-9
2	Feng Y. et al. (2025)	6/6	SD rats	AIA	2	Subcutaneous injection	15 d	Arthritis index, paw volumes, IL-6, IL-1β
3	Liu et al. (2024)	5/5	DBA/1 mice	CIA	50/100	Intraperitoneal injection	35 d	Arthritis index, IL-1β, IL-6, TNF-α
4	Li et al. (2024)	6/6	SD rats	AA	25/50	Oral gavage	21 d	TNF-α, IL-6, IL-1β, IL-10, RANKL, OPG, MMP-9
5	Li R. et al. (2023)	6/6	Rat	AIA	25/50/100	Intraperitoneal injection	30 d	Arthritis index, paw volumes, TNF-α, IL-1β, IL-6
6	Li J. et al. (2023)	11/11	DBA/1 mice	CIA	25/50/100	Intraperitoneal injection	30 d	Arthritis index, TNF-α, IL-1β, IL-6
7	Jiang Z. et al. (2023)	12/12	Wistar rats	CIA	30/120/240	Oral gavage	28 d	Arthritis index, paw volumes, IL-6, TNF-α, histological score
8	Jiang H et al. (2023)	10/10	C57BL/6 mice	AA	90	Oral gavage	30 d	Arthritis index, TNF-α, IL-10, IL-6
9	Yi et al. (2021)	8/8	SD rats	AIA	120	Oral gavage	30 d	Arthritis index, paw volumes, TNF-α
10	Liao et al. (2021)	11/11	DBA/1 mice	CIA	25/50/100	Intraperitoneal injection	21 d	Arthritis index, IL-6, IL-1β, TNF-α, histological scores
11	Chen et al. (2021)	10/10	SD rats	AIA	60	Oral gavage	16 d	Paw volumes, TNF-α, IL-1β, IL-6
12	Xu et al. (2018)	10/10	SD rats	CIA	25	Oral gavage	19 d	Arthritis index, TNF-α, IL-6, IL-1β, MMP-9
13	Qian et al. (2018)	10/10	SD rats	CIA	30/60/120	Oral gavage	28 d	Arthritis index, histological score
14	Liu et al. (2018)	6/6	DBA/1 mice	CIA	50/100	Oral gavage	20 d	Arthritis index, paw volumes, IL-6, IL-1β, TNF-α, IL-10
15	Tong et al. (2016)	8/8	DBA/1 mice	CIA	120	Oral gavage	14 d	Arthritis index, IL-1β, TNF-α, IL-6, IL-10
16	Tong et al. (2015)	8/8	Wistar rats	CIA	20/60/120	Oral gavage/intraperitoneal injection	14 d	Arthritis index, paw volumes, IL-1β, TNF-α, IL-6, IL-10, histological scores
17	Sun et al. (2014)	7/7	SD rats	CIA	120	Oral gavage	37 d	Arthritis index, IL-6, histological scores, RANKL, OPG
18	Mu et al. (2013)	10/10	SD rats	AIA	100	Oral gavage	23 d	Arthritis index, TNF-α, IL-6, IL-1β
19	Zhou et al. (2008)	10/10	Wistar rats	CIA	25/50/100	Intraperitoneal injection	30 d	Arthritis index, paw volumes, IL-1β, IL-6, MMP-9
20	Xu et al. (2025)	10/10	Wistar rats	AA	100/200/400	Oral gavage	28 d	Arthritis index, TNF-α, IL-6, IL-1β
21	Feng X. et al. (2025)	6/6	Wistar rats	CIA	100	Oral gavage	28 d	Arthritis index, paw volumes, TNF-α, IL-1β, IL-6, IL-10
22	Wen et al. (2024)	8/8	SD rats	CIA	0.375	Intra-articular injection	28 d	Arthritis index, TNF-α, IL-6, IL-1β
23	Shang (2024)	6/6	SD rats	CIA	0.25	Intra-articular injection	8 d	Arthritis index, TNF-α, IL-1β
24	Huang et al. (2023)	10/10	Wistar rats	AA	100/200/400	Oral gavage	16 d	Arthritis index, paw volumes, TNF-α, IL-6, IL-1β
25	Zhang et al. (2022)	5/5	SD rats	AIA	1.2 animal/week/18	Oral gavage/intra-articular injection	28 d	Arthritis index, TNF-α, IL-1β, IL-6
26	Hu et al. (2022)	5/5	C57BL/6 mice	CIA	100	Intraperitoneal injection	33 d	Arthritis index, TNF-α, IL-6, histological scores
27	Ji (2021)	8/8	C57BL/6 mice	CIA	30/300	Oral gavage	14 d	Arthritis index, MMP-9
28	Zhao et al. (2018)	16/16	SD rats	AIA	30/60/120	Oral gavage	21 d	IL-1β, IL-6, IL-10, histological scores
29	Xu (2018)	8/8	DBA/1 mice	CIA	100	Oral gavage	21 d	Arthritis index, TNF-α
30	Zhou (2016)	8/8	SD rats	AIA	40/80/120	Oral gavage	36 d	Arthritis index, paw volumes, TNF-α, IL-6
31	Zhang (2016)	10/10	DBA/1 mice	CIA	100	Oral gavage	20 d	Arthritis index, TNF-α
32	Gu (2016)	10/10	Wistar rats	CIA	50	Oral gavage	31 d	Arthritis index, paw volumes, TNF-α, IL-1β, histological scores, MMP-9
33	Xu (2015)	9/9	SD rats	CIA	120	Oral gavage	37 d	Arthritis index, RANKL, OPG
34	Zhang (2013)	8/8	SD rats	CIA	60/120	Oral gavage	31 d	Arthritis index, IL-6, RANKL, OPG
35	Wang et al. (2012)	10/10	Wistar rats	AA	4	Oral gavage	28 d	Arthritis index, paw volumes, TNF-α, histological scores
36	Ding (2012)	7/7	SD rats	CIA	120	Oral gavage	38 d	Arthritis index, IL-6, RANKL, OPG
37	Chen (2011)	10/10	Wistar rats	AA	40	Oral gavage	28 d	Arthritis index, IL-6, histological scores
38	Yang et al. (2006)	12/12	Wistar rats	AA	30/60/120	NR	21 d	Arthritis index, TNF-α, IL-1β, IL-10

Abbreviations: Sin, Sinomenine; SD, Sprague-Dawley; NR, no reports; CIA, Collagen-Induced Arthritis; AIA, Adjuvant-Induced Arthritis; AA, Antigen-Induced Arthritis; TNF-α, Tumor Necrosis Factor-α; IL-1β, Interleukin-1β; IL-6, Interleukin-6; IL-10, Interleukin-10; MMP-9, Matrix Metalloproteinase-9; RANKL, Receptor Activator of Nuclear Factor-κB ligand; OPG, osteoprotegerin.

### 3.2 Risk of bias assessment

**FIGURE 2:**
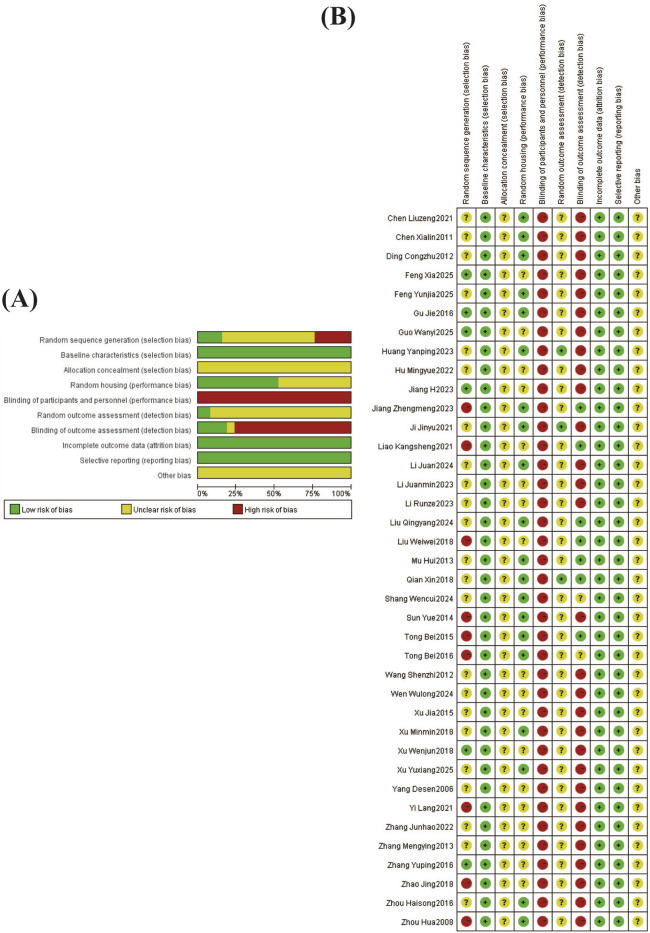
The risk of bias assessment of the 38 studies included in this meta-analysis based on SYRCLE’s risk of bias tool. Note: **(A)** Risk of bias graph; **(B)** Risk of bias summary.

The quality risk assessment results are presented in (Figure 2), Twenty-nine studies referenced a “random number table” or indicated random allocation without detailing the specific procedure. All included studies reported similar baseline characteristics between groups, but none described the method of allocation concealment. Eighteen studies did not specify whether animals were randomly housed. Regarding blinding, none of the included studies reported blinding of participants and personnel, while seven studies reported blinding of outcome assessment. Only three studies described whether animals were randomly selected for outcome assessment. All studies demonstrated complete outcome data and reported the prespecified outcomes. No study clearly stated other potential risks of bias.

### 3.3 Meta-analysis

#### 3.3.1 Clinical joint manifestations

**FIGURE 3:**
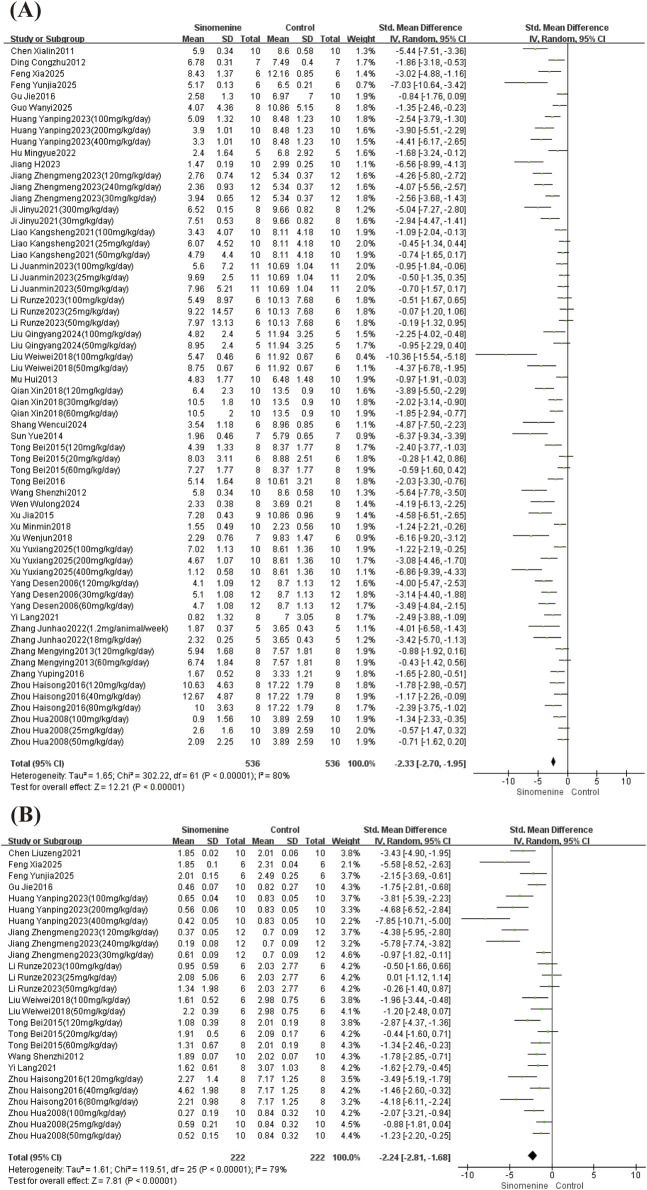
Arthritis index and Paw volume forest plot. Note: **(A)** Arthritis index forest plot; **(B)** Paw volume forest plot.

This study analyzed the arthritis index and paw volume. A total of 35 studies reported the arthritis index, and 13 studies reported paw volume. SMDs were calculated using the Hedges’ g method. Due to significant heterogeneity in both the arthritis index (I^2^ = 80%, P < 0.00001) and paw volume (I^2^ = 79%, P < 0.00001), random-effects models were applied. Compared with controls, sinomenine significantly reduced the arthritis index (SMD = −2.33, 95% CI: −2.70 to −1.95; *P* < 0.00001) (Figure 3A) and paw volume (SMD = −2.24, 95% CI: −2.81 to −1.68; *P* < 0.00001) (Figure 3B).

Subgroup analyses were performed for both outcomes. For the arthritis index, considerable heterogeneity persisted within each subgroup when stratified by dose or by intervention duration. However, high-dose sinomenine showed a stronger therapeutic effect in reducing the arthritis index (SMD = −3.40, 95% CI: −4.10 to −2.69; *P* < 0.00001). Furthermore, both the intraperitoneal and intra-articular injection subgroups showed very low heterogeneity within their groups (I^2^ = 0%), indicating that their efficacy was highly consistent and reproducible. In contrast, the oral gavage subgroup was effective (SMD = −2.81, 95% CI: −3.30 to −2.32; *P* < 0.00001), but there was considerable heterogeneity (I^2^ = 78%). Subcutaneous injection was not analyzed as only one study was available. Combined with the significant test for subgroup differences (I^2^ = 96.5%, *P* < 0.00001), this indicates that the administration route is a key factor contributing to the overall heterogeneity in the arthritis index group.

For paw volume, within-subgroup heterogeneity did not differ significantly by intervention duration. However, both dose and administration route partially explain the heterogeneity (test for subgroup differences: I^2^ = 91.6%, *P* < 0.00001). The high-dose subgroup showed the largest mean effect but also considerable heterogeneity (I^2^ = 78%), whereas the low-dose subgroup had a more modest effect with the greatest consistency across studies (I^2^ = 20%). A dose-dependent effect was observed: the pooled effect size increased with dose. Furthermore, intraperitoneal injection yielded consistent results (I^2^ = 34%), while oral gavage, though effective (SMD = −2.93, 95% CI: −3.65 to −2.22; *P* < 0.00001), was associated with high heterogeneity (I^2^ = 78%). The forest plots for all subgroup analyses are provided in the (Supplementary Figures S1–S6).

#### 3.3.2 Anti-inflammatory indicators

**FIGURE 4:**
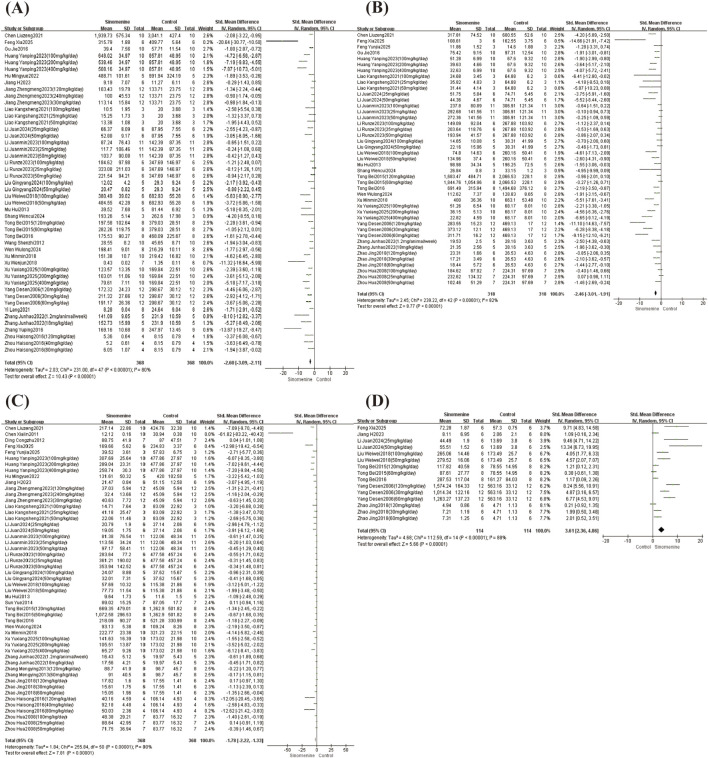
TNF-α, IL-1β, IL-6 and IL-10 forest plot. Note: **(A)** TNF-α forest plot; **(B)** IL-1β forest plot; **(C)** IL-6 forest plot; **(D)** IL-10 forest plot.

Among the included studies, TNF-α levels were analyzed in 27 studies, IL-1β in 22 studies, IL-6 in 27 studies, and IL-10 in 8 studies. There was high heterogeneity between these articles, TNF-α (I^2^ = 80%, *P* < 0.00001); IL-1β (I^2^ = 82%, *P* < 0.00001); IL-6 (I^2^ = 80%, *P* < 0.00001); IL-10 (I^2^ = 88%, *P* < 0.00001), random-effects models were applied. Compared with controls, sinomenine significantly reduced the levels of TNF-α (SMD = −2.60, 95% CI: −3.09 to −2.11; *P* < 0.00001), IL-1β (SMD = −2.46, 95% CI: −3.01 to −1.91; *P* < 0.00001) and IL-6 (SMD = −1.78, 95% CI: −2.22 to −1.33; *P* < 0.00001) (Figures 4A–C). In contrast, IL-10 levels were significantly increased (SMD = 3.61, 95% CI: 2.36 to 4.86; *P* < 0.00001) (Figure 4D).

Subgroup analyses were performed for all outcomes, except for the analysis of administration route in the IL-10 group, as this method was uniformly oral gavage across all included studies. For the TNF-α group, considerable heterogeneity persisted within each subgroup when stratified by dose or by intervention duration. However, it was observed that high-dose (SMD = −3.25, 95% CI: −4.35 to −2.16; *P* < 0.00001) and short-term (SMD = −3.75, 95% CI: −4.61 to −2.89; *P* < 0.00001) sinomenine showed a stronger treatment effect on reducing the TNF-α content. Administration route is a key factor contributing to the overall heterogeneity in the TNF-α group, intraperitoneal injection yielded consistent results (I^2^ = 0%), whereas oral gavage (SMD = −3.19, 95% CI: −3.86 to −2.52; *P* < 0.00001) and intra-articular injection (SMD = −4.15, 95% CI: −7.89 to −0.42; *P* = 0.03), while effective, showed considerable heterogeneity (I^2^ = 81%; I^2^ = 72%, respectively).

For both IL-1β and IL-6 groups, considerable heterogeneity persisted across subgroups stratified by dose or intervention duration. However, short-term sinomenine treatment was associated with a greater reduction in the levels of both IL-1β (SMD = −3.54, 95% CI: −4.45 to −2.63; *P* < 0.00001) and IL-6 (SMD = −2.86, 95% CI: −3.76 to −1.96; *P* < 0.00001). Furthermore, the administration route may be a key factor contributing to the overall heterogeneity. In the analysis of IL-1β, both the intraperitoneal and intra-articular injection subgroups showed low heterogeneity (I^2^ = 14% and I^2^ = 0%, respectively), indicating highly consistent efficacy. In contrast, the oral gavage subgroup was effective (SMD = −2.84, 95% CI: −3.33 to −2.15; *P* < 0.00001) but exhibited high heterogeneity (I^2^ = 77%). Subcutaneous injection was not analyzed as only one study was available. A similar pattern was observed for IL-6. The intraperitoneal injection subgroup exhibited low heterogeneity (I^2^ = 10%) and consistent efficacy. In contrast, the oral gavage subgroup was effective (SMD = −2.42, 95% CI: −3.09 to −1.75; *P* < 0.00001) but highly heterogeneity (I^2^ = 85%). No statistically significant effects were observed in either the intra-articular or subcutaneous injection groups.

For the IL-10 group, we found no significant change in the heterogeneity when stratified by dose or by intervention duration, possibly due to variations in rat strain or modeling method; no significant differences were observed between these subgroups (I^2^ = 46.1%, *P* = 0.16; I^2^ = 0%, *P* = 0.74). The forest plots for all subgroup analyses are provided in the (Supplementary Figures S7–S17).

#### 3.3.3 Joint-protective indicators

**FIGURE 5:**
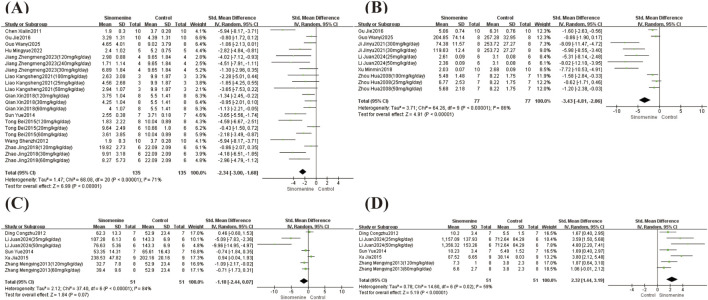
Histological score, MMP-9, RANKL and OPG forest plot. Note: **(A)** Histological score forest plot; **(B)** MMP-9 forest plot; **(C)** RANKL forest plot; **(D)** OPG forest plot.

Among the included studies, histological scores were analyzed in 11 studies, MMP-9 content in 6 studies, and both RANKL and OPG levels in 5 studies. Large heterogeneity was observed across these studies, histological score (I^2^ = 71%, *P* < 0.00001); MMP-9 (I^2^ = 86%, *P* < 0.00001); RANKL (I^2^ = 84%, *P* < 0.00001); OPG (I^2^ = 59%, *P* = 0.02), random-effects models were applied. Compared with controls, sinomenine significantly reduced the histological scores (SMD = −2.34, 95% CI: −3.00 to −1.68; *P* < 0.00001) and MMP-9 levels (SMD = −3.43, 95% CI: −4.81 to −2.06; *P* < 0.00001) (Figures 5A,B); RANKL levels showed no statistically significant change (SMD = −1.18, 95% CI: −2.44 to 0.07; *P* = 0.07) (Figure 5C), and the content of OPG increased significantly (SMD = 2.32, 95% CI: 1.44 to 3.19; *P* < 0.00001) (Figure 5D).

Subgroup analyses were performed for all outcomes, except for the analysis of administration route in the RANKL and OPG groups, as all studies in these groups uniformly used oral gavage. For the histological score, the test for differences between subgroups indicated that dose (I^2^ = 17%, *P* = 0.30), intervention duration (I^2^ = 0%, *P* = 0.46), and administration route (I^2^ = 0%, *P* = 0.33) were not major sources of the overall heterogeneity. However, the medium-dose subgroup (I^2^ = 18%, *P* = 0.29) and intraperitoneal administration (I^2^ = 38%, *P* = 0.17) showed lower heterogeneity, suggesting more consistent effects under these specific conditions. For the MMP-9 group, dose, intervention duration, and administration route partially explain the heterogeneity (test for subgroup differences: I^2^ = 90.2%, *P* < 0.00001; I^2^ = 87.2%, *P* = 0.005; I^2^ = 90.6%, *P* = 0.001, respectively). The medium-dose, long-term, and intraperitoneal injection subgroups showed consistent results with no heterogeneity (I^2^ = 0%). In contrast, although the low-dose (SMD = −3.82, 95% CI: −5.66 to −1.99; *P* < 0.00001), short-term (SMD = −5.83, 95% CI: −9.00 to −2.65; *P* = 0.00003), and oral gavage (SMD = −5.01, 95% CI: −7.27 to −2.75; *P* < 0.00001) subgroups each demonstrated significant reductions in MMP-9, they were associated with considerable heterogeneity (I^2^ = 87%, 90%, and 89%, respectively). High-dose subgroup was not analyzed due to only one available study.

For the RANKL group, although the overall effect was not statistically significant and considerable heterogeneity persisted across subgroups stratified by dose or intervention duration, subgroup analysis indicated that low-dose and short-term sinomenine showed a stronger treatment effect on reducing the RANKL content (SMD = −7.06, 95% CI: −11.74 to −2.38; *P* = 0.003). For the OPG group, we found that stratifying by either dose or intervention duration significantly reduced within-group heterogeneity, indicating that both factors are important sources of variability. Furthermore, subgroup analysis indicated that low-dose and short-term sinomenine treatment had a better therapeutic effect on increasing the OPG content (SMD = 4.06, 95% CI: 2.44 to 5.69; *P* < 0.00001). The leave-one-out sensitivity analysis found that after the exclusion of one study (Xu, 2015), the heterogeneity decreased (I^2^ = 49%, *P* = 0.08). A review of the full text suggested that the heterogeneity might be due to the use of a sustained-release sinomenine suspension in that study. The pharmacokinetic characteristics of this formulation, particularly in terms of onset of action and maintenance of plasma concentration, could differ from those of the conventional preparations used in other studies (De Haan and Lerk, 1984). The forest plots for all subgroup analyses are provided in the (Supplementary Figures S18–S27).

#### 3.3.4 Other indicators

The impact of sinomenine on liver safety was assessed via serum alanine aminotransferase (ALT) and aspartate aminotransferase (AST) levels. Pooled results from four studies showed that sinomenine significantly reduced ALT levels (SMD = −0.90; 95% CI: −1.47 to −0.32; *P* = 0.002), with moderate heterogeneity (I^2^ = 52%). Correspondingly, the meta-analysis of AST showed no significant overall effect (SMD = 0.18; 95% CI: −0.85 to 1.20; *P* = 0.73), with high heterogeneity (I^2^ = 73%). The forest plots for ALT and AST are provided in the (Supplementary Figure S28). Furthermore, a review of other safety-related indicators across the 38 included studies showed that body weight was reported in 6 studies, major organ histopathology in 3 studies, immune organ indices in 4 studies, and myelosuppression parameters in 2 studies. No consistent evidence of adverse effects was observed at therapeutic doses. Regarding dose-dependent signals, one study noted that high-dose sinomenine (120 mg/kg) may cause liver injury in rats (Zhao et al., 2018), and another study mentioned that higher doses might increase the risk of allergic reactions (Guo et al., 2025). No other dose-associated safety signals were consistently reported (Supplementary Table S2).

**FIGURE 6:**
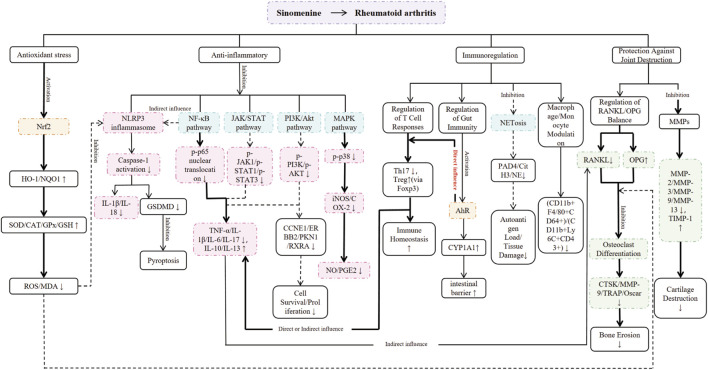
The mechanism of sinomenine in treating RA. Note: The colour area is the outcome index. Blue represents the pro-RA pathway, orange represents the anti-RA pathway, red represents inflammation, and green represents joint protection. To clearly distinguish the evidentiary basis for each pathway, a visual coding system has been implemented. Thick solid lines represent well-established mechanisms (Level A), which are supported by numerous independent studies. Normal solid lines indicate pathways with strong supporting evidence (Level B), confirmed by several studies. Dashed lines are used for speculative or preliminary pathways (Level C), which are proposed by single studies and warrant further investigation. A detailed breakdown of this evidence grading is provided in (Supplementary Table S3).

Besides the measures already discussed, we found other outcome indicators that may help confirm the treatment effects of sinomenine on RA. The mechanism of action of these indicators has been summarized in (Figure 6).

#### 3.3.5 Publication bias

**FIGURE 7:**
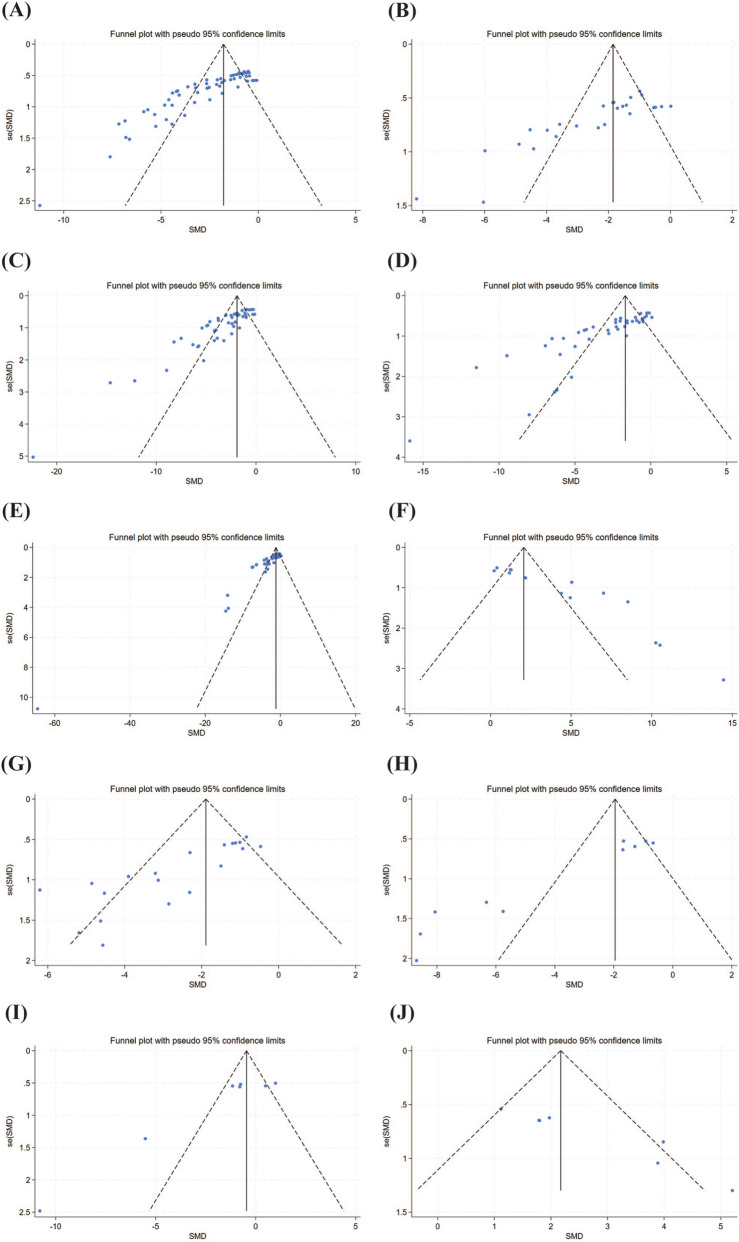
Outcome Index Funnel Plot. Note: **(A)** Arthritis index funnel plot; **(B)** Paw volume funnel plot; **(C)** TNF-α content funnel plot; **(D)** IL-1β content funnel plot; **(E)** IL-6 content funnel plot; **(F)** IL-10 content funnel plot; **(G)** Histological score funnel plot; **(H)** MMP-9 content funnel plot; **(I)** RANKL content funnel plot; **(J)** OPG content funnel plot.

For results with significant heterogeneity, publication bias was assessed using funnel plots (Figure 7) and Egger’s test (Table 2), revealing significant bias. This is likely because all included studies were animal experiments, most of which reported positive findings.

**TABLE 2:** Egger test.

Number	Study	t	P	95% Conf. interval	
1	Arthritis index	−14.22	0.000	−7.417109	−5.588109
2	Paw volume	−7.84	0.000	−9.290418	−5.419103
3	TNF-α	−10.75	0.000	−5.757301	−3.941053
4	IL-1β	−9.89	0.000	−6.637723	−4.387196
5	IL-6	−11.08	0.000	−6.148331	−4.260361
6	IL-10	8.28	0.000	4.665569	7.958486
7	Histological score	−6.83	0.000	−5.967155	−3.169279
8	MMP-9	−10.80	0.000	−7.535583	−4.884735
9	RANKL	−3.93	0.011	−9.809952	−2.054649
10	OPG	6.81	0.001	3.615471	8.002688

#### 3.3.6 Sensitivity analysis

**FIGURE 8:**
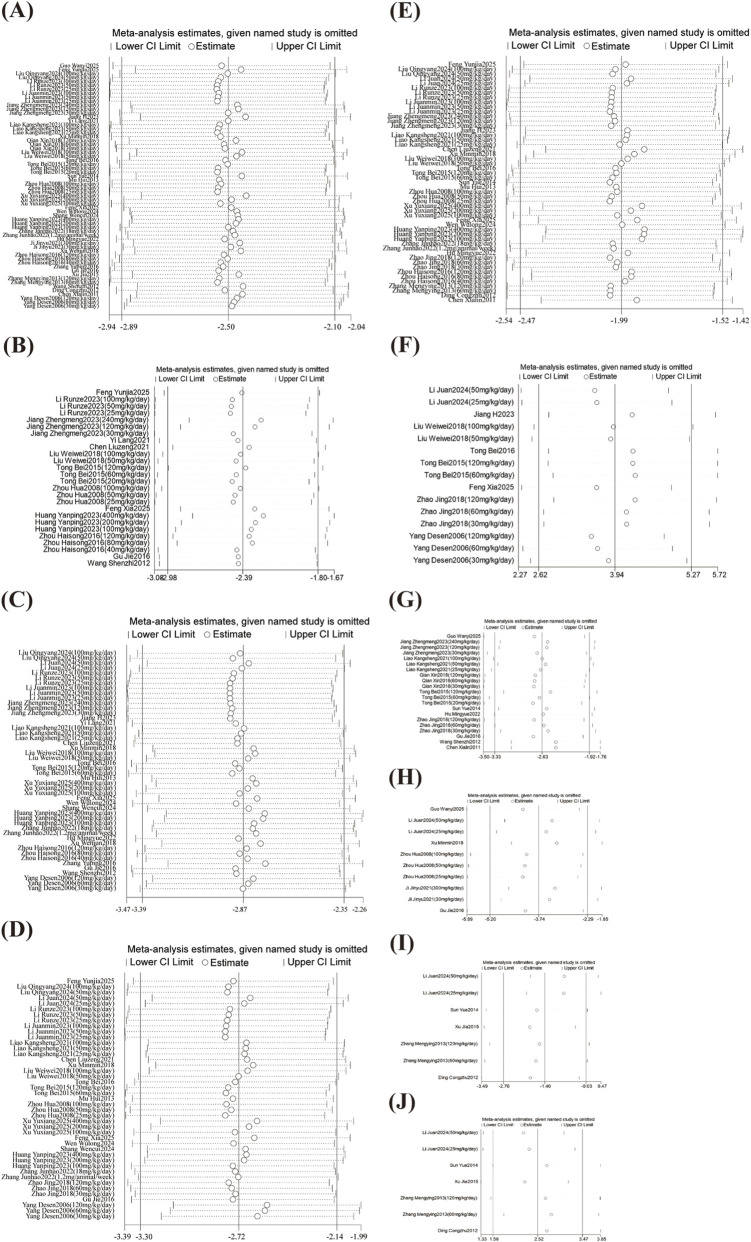
Outcome indicators sensitivity analysis. Note: **(A)** Arthritis index sensitivity analysis; **(B)** Paw volume sensitivity analysis; **(C)** TNF-α content sensitivity analysis; **(D)** IL-1β content sensitivity analysis; **(E)** IL-6 content sensitivity analysis; **(F)** IL-10 content sensitivity analysis; **(G)** Histological score sensitivity analysis; **(H)** MMP-9 content sensitivity analysis; **(I)** RANKL content sensitivity analysis; **(J)** OPG content sensitivity analysis.

Sensitivity analysis was conducted by removing each study in turn and comparing the recalculated pooled effect size with the original. As shown in (Figure 8), no significant differences were observed for any outcome, indicating that the overall results are robust and reliable. To further assess potential data integrity concerns, we performed Granularity-Related Inconsistency of Means (GRIM) testing on integer outcomes reported numerically (arthritis index and histological scores). Among 11 such studies, seven showed potential inconsistencies (Supplementary Table S4). Excluding these seven studies individually or simultaneously did not materially alter the pooled estimates (Supplementary Figures S29,S30). Additionally, basic visual inspection of all figures in the included studies revealed no clear evidence of image manipulation.

## 4 Discussion

In 2020, musculoskeletal disorders ranked as the second leading cause of non-fatal disability worldwide, and RA constituted a major and rising contributor (Yin et al., 2025; Chen et al., 2024). The worldwide prevalence of RA is increasing steadily, and model-based projections indicate the number of affected individuals may approach 31.7 million by 2050. It causes long-term disability and increased risk of premature death, and patients face escalating physical, mental, and social burdens (Black et al., 2023). The disease mechanisms are not fully understood, involving autoimmune dysregulation, chronic synovitis, inflammatory networks, fibroblast activation, bone and cartilage destruction, and immune-bone crosstalk (Gravallese and Firestein, 2023; Catrina et al., 2016). Among these, dysregulated inflammatory responses and impaired bone protection mechanisms are central pathological focuses in current research.

Studies indicate that the immune-inflammatory response is a central driver of RA pathology, leading to pathological hyperplasia and invasion of the synovial tissue. Dysfunction of the synovial membrane and infiltration of immune cells are typical pathological manifestations of RA. The characteristic is that pro-inflammatory cytokines (such as IL-1β, IL-6, IL-17, and TNF-α) are significantly upregulated in the early stage of the disease, while anti-inflammatory mediators such as IL-10 are relatively insufficient or have impaired responses (Muheremu et al., 2025). This imbalance of cytokines exacerbates local inflammation and tissue damage. Under the persistent stimulation of this inflammatory environment, synovial fibroblasts become activated and undergo phenotypic transformation, gaining invasive properties, which directly contribute to the subsequent destruction of joint structures (Nygaard and Firestein, 2020).

Progressive destruction of bone and cartilage is the core pathological process leading to disability in RA. This structural damage is driven by an imbalance in osteoclast-osteoblast coupling and excessive degradation of the extracellular matrix (McInnes and Schett et al., 2011). Histological scoring is a classic indicator for evaluating the overall degree of joint damage, which directly reflects the total pathological burden of the joint by systematically quantifying synovial hyperplasia, inflammatory infiltration, pannus formation, and bone erosion.

At the molecular level, MMP-9, a key protease, is significantly elevated in RA synovium (Stojanovic et al., 2023). By directly breaking down major cartilage components such as type II collagen and proteoglycans, it acts as a critical mediator of cartilage destruction and joint space narrowing (Grillet et al., 2021). Additionally, the imbalance of bone metabolism is at the core of bone erosion, mainly regulated by a disrupted ratio between RANKL and its natural decoy receptor OPG. Activated immune cells and synovial fibroblasts overexpress RANKL, which binds to RANK on osteoclast precursors, strongly driving their differentiation and activation. In contrast, insufficient OPG expression fails to effectively neutralize RANKL, leading to overactive osteoclast function. This cascade reaction results in progressive bone resorption and erosion, ultimately causing irreversible damage to the joint structure (Komatsu and Takayanagi, 2022).

Sinomenine is a natural active metabolite derived from plants. Both preclinical and clinical studies have confirmed its therapeutic effect on RA (Li et al., 2024; Shi et al., 2022). Mechanistically, it produces immunomodulatory, anti-inflammatory, and joint-protective effects by regulating key pathways including NF-κB, MAPK, and JAK/STAT, thereby restoring immune homeostasis, inhibiting pro-inflammatory cytokines, and reducing synovial hyperplasia and bone erosion (Hou et al., 2024; Jiang et al., 2024). Sinomenine also alleviates RA-related pain and mechanical hypersensitivity, which may be related to its regulation of L-type calcium channels, monoaminergic systems, and N-methyl-D-aspartate (NMDA) receptors (Lagerström, 2015). In clinical practice, compared to the combination of methotrexate (MTX) and leflunomide (LEF), the combination of sinomenine and MTX can reduce gastrointestinal side effects and hepatotoxicity (Huang et al., 2019). These findings support the therapeutic potential of sinomenine.

During the process of conducting this meta-analysis, we found that sinomenine exerts its therapeutic effects through a multi-target mechanism. It activates the Nrf2 pathway, enhances the antioxidant system and reduces the level of reactive oxygen species (ROS). Since ROS is a key activator of the NOD-like receptor thermal protein domain associated protein 3 (NLRP3) inflammasome, this reduction indirectly inhibits NLRP3 activation, thereby suppressing the cleavage of gasdermin D (GSDMD) and the release of pro-inflammatory cytokines downstream. In addition, sinomenine simultaneously inhibits multiple inflammatory pathways, including NF-κB. These pathways interact with each other, jointly suppressing pro-inflammatory factors and promoting anti-inflammatory factors. Notably, inhibiting NF-κB also indirectly limits the activation of the NLRP3, forming a multi-layered and reinforced anti-inflammatory network. Furthermore, sinomenine can also regulate systemic and intestinal immunity. It activates the aryl hydrocarbon receptor (AhR), which plays a dual role in regulating T cell responses and enhancing intestinal barrier function. Sinomenine also inhibits neutrophil extracellular trap (NET) formation (NETosis), thereby reducing the self-antigen load. These effects collectively assist in restoring immune homeostasis.

**FIGURE 9:**
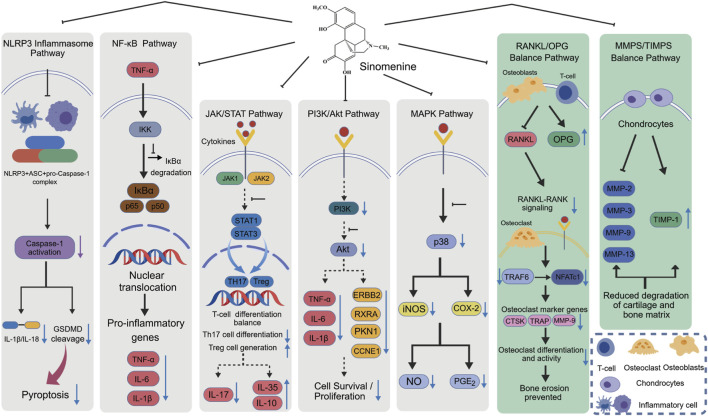
Schematic diagram of the potential mechanisms of Sinomenine in the treatment of RA. See Figure 6 for line styles indicating evidence levels. This diagram illustrates the dual anti-inflammatory and bone-protective effects of Sinomenine, which are exerted through multiple targets and pathways. For its anti-inflammatory effects, Sinomenine can inhibit the NLRP3 inflammasome, Nuclear factor-kappa B (NF-κB), Janus kinase/signal transducer and activator of transcription (JAK/STAT), Phosphatidylinositol 3-kinase/protein kinase B (PI3K/Akt), and Mitogen-activated protein kinase (MAPK) signaling pathways. This leads to a reduction in the production and release of pro-inflammatory cytokines (e.g., TNF-α, IL-1β, IL-6, IL-17), regulates the balance of T-cell subsets, and ultimately suppresses the inflammatory response. For its bone-protective effects, Sinomenine upregulates OPG and downregulates the RANKL. It also modulates the balance between MMPs and their TIMPs. These actions effectively inhibit osteoclast activity and cartilage matrix degradation, thereby preventing joint bone erosion and destruction. The figure was created with BioGDP.com (Jiang et al., 2025).

The combined effects described above work together to protect joint structures. They help correct the RANKL/OPG imbalance, which inhibits osteoclast differentiation and bone erosion. Sinomenine also suppresses the excessive expression of matrix metalloproteinases (MMPs) and promotes the generation of tissue inhibitors of metalloproteinases (TIMPs), thereby restoring the MMP/TIMP balance and directly helping to maintain cartilage integrity. Through these multi-target actions, sinomenine simultaneously targets synovial inflammation and overall immune dysregulation, effectively breaking the damaging cycle of inflammation, oxidative stress, and joint destruction in RA. Overall, although sinomenine exerts broad mechanisms of action, its ultimate therapeutic effects primarily manifest in two core aspects: potent anti-inflammatory activity and direct joint protection, with key pathways illustrated in (Figure 9).

The subgroup analysis in this meta-analysis revealed different therapeutic characteristics. The high-dose sinomenine could more stably improve the clinical joint manifestations and inflammatory indicators, which might be related to its broad and potent suppression of NF-κB and cytokines such as TNF-α. In contrast, the low-dose treatment showed better regulatory effects on joint protection indicators. This effect may come from a more selective inhibition of NF-κB, which effectively corrects the RANKL/OPG imbalance. The short-term administration effect was particularly effective, which might be consistent with its pharmacodynamic characteristics of rapidly inhibiting NF-κB, reducing cytokines (TNF-α, IL-1β, IL-6) and inhibiting destructive mediators (MMP-9, RANKL). Analysis of administration routes showed that intraperitoneal injection produced highly consistent results (I^2^ = 0%), providing the strongest preclinical evidence for efficacy, likely due to its more predictable bioavailability and direct systemic exposure. Oral administration was also effective but showed significant heterogeneity in results, which could stem from variable intestinal absorption and first-pass metabolism inherent to this route. In terms of safety, sinomenine did not cause significant liver cell injury in the included studies, and our broader review of the included literature revealed no consistent evidence of myelosuppression or major organ damage at therapeutic doses. This suggests a favorable preclinical safety profile compared to some conventional DMARDs. However, these findings are based on a limited number of studies, and when considering the benefit-risk profile, it is important to note that some studies allude to dose-dependent adverse effects, such as a higher risk of allergic reactions at the high doses sometimes required for clinical efficacy. Additionally, given its nature as a morphinan derivative and the associated potential nervous system effects, further studies are warranted to fully establish its overall safety profile.

## 5 Conclusion

Through quantitative synthesis, this study first systematically verified the dual anti-inflammatory and joint-protective mechanisms of sinomenine in RA rodent models. Results show that sinomenine significantly alleviates systemic inflammation and mitigates joint damage compared to controls. Subgroup analyses further indicate that its efficacy is influenced by dose, intervention duration, and administration route, providing experimental evidence for the key parameters in clinical protocol design. Although other potential mechanisms, such as antioxidative stress, were not taken into consideration in this analysis due to variability in research designs, the existing evidence points to a multidimensional therapeutic potential for sinomenine in modulating RA pathology.

However, this meta-analysis has several limitations. In terms of methodology, these limitations exist both in the original studies and in our review process. Some of the original studies were considered to have a relatively high risk of bias in the areas of selection, performance, and detection, mainly due to a lack of detail on randomization and blinding implementation. Additionally, our analysis may be influenced by the restriction of literature searches to Chinese and English databases, the inherent subjective judgment involved in data extraction, and a potential publication bias favoring studies with positive outcomes. Significant heterogeneity in some outcome indicators across studies should also be considered. Although subgroup analyses have explored the effects of dose, administration route, and intervention duration, differences in animal strains, study model types, and detection methods may still affect the consistency of the results. In terms of data availability, our analysis was constrained by the outcomes reported in the primary literature. Only three of the included studies reported bone-related parameters (e.g., micro-CT), limiting a systematic assessment of the effect of sinomenine on joint integrity and reflecting a notable evidence gap. It should be noted that current animal models are difficult to fully simulate the complex pathological features of human RA. This species difference directly restricts the effective translation of preclinical research findings into clinical practice. This study confirmed that sinomenine improves systemic inflammation and joint damage in RA rodent models through anti-inflammatory and joint-protection mechanisms, and provided a reference for treatment optimization. However, considering the above limitations, its ultimate clinical application still needs to be verified through high-quality clinical trials. Therefore, future clinical trials are crucial to validate these findings, and most importantly, to establish an optimal therapeutic window by carefully evaluating different doses, durations, and administration routes, thereby paving the way for the safe and effective application of sinomenine in RA treatment.

## Data Availability

The datasets presented in this study can be found in online repositories. The names of the repository/repositories and accession number(s) can be found in the article/Supplementary Material.
