# Supplementary material for: Sinomenine ameliorates rheumatoid arthritis in rodent models: a systematic review and meta-analysis of anti-inflammatory and joint-protective effects
Source: Front Pharmacol. 2026 May 4;17:1800265. doi: 10.3389/fphar.2026.1800265 (PMC13180913; doi:10.3389/fphar.2026.1800265)
Supplement: Supplementary file 1 [file Supplementaryfile1.docx]

**Supplementary Material**

**Sinomenine Ameliorates Rheumatoid Arthritis in Rodent Models: A Systematic Review and Meta-Analysis of Anti-Inflammatory and Joint-Protective Effects**

**File S1.** Search strategy.

**Search Strategy in PubMed**

| **Steps** | **Search terms** | **Results** |
| --- | --- | --- |
| #1 | "sinomenine" [Supplementary Concept] | 511 |
| #2 | (((sinomenine[All Fields]) OR (cucoline[All Fields])) OR (sinomenium actum[All Fields])) | 725 |
| #3 | Search #1 OR #2 | 725 |
| #4 | "Rheumatoid Arthritis"[Mesh Terms] | 133788 |
| #5 | (((Rheumatoid Arthritis[All Fields]) OR ( Rheumatoid[All Fields])) OR (Arthritis[All Fields])) | 438191 |
| #6 | Search #4 OR #5 | 438191 |
| #7 | Search #3 AND #6 | 212 |

**Search Strategy in Web of Science**

| **Steps** | **Search terms** | **Results** |
| --- | --- | --- |
| #1 | (ALL=(sinomenine OR cucoline OR sinomenium actum)) | 786 |
| #2 | ALL=(Rheumatoid Arthritis OR Rheumatoid OR Arthritis) | 356491 |
| #3 | #1 AND #2 | 250 |

**Search Strategy in Embase**

| **Steps** | **Search terms** | **Results** |
| --- | --- | --- |
| #1 | 'sinomenine'/exp OR 'sinomenine' | 1308 |
| #2 | 'cucoline':ab,ti OR 'sinomenium actum':ab,ti | 3 |
| #3 | Search #1 OR #2 | 1310 |
| #4 | rheumatoid AND ('arthritis'/exp OR arthritis) | 314900 |
| #5 | 'Rheumatoid':ab,ti OR 'Arthritis':ab,ti | 365062 |
| #6 | Search #4 OR #5 | 437331 |
| #7 | Search #3 AND #6 | 328 |

**Search Strategy in Cochrane Library**

| **Steps** | **Search terms** | **Results** |
| --- | --- | --- |
| #1 | (sinomenine):ti,ab,kw OR (cucoline):ti,ab,kw OR (sinomenium actum):ti,ab,kw | 20 |
| #2 | MeSH descriptor: [Arthritis, Rheumatoid] explode all trees | 8214 |
| #3 | (Rheumatoid):ti,ab,kw OR (Arthritis):ti,ab,kw | 29978 |
| #4 | Search #2 OR #3 | 30383 |
| #5 | Search #1 AND #4 | 12 |

**Search Strategy in CNKI**

| **Steps** | **Search terms** | **Results** |
| --- | --- | --- |
| #1 | ((SU%=青藤碱 OR SU%=青风藤碱 OR SU%=青风藤) OR (TKA=青藤碱 OR TKA=青风藤碱 OR TKA=青风藤)) AND ((SU%=类风湿关节炎 OR SU%=类风湿性关节炎 OR SU%=类风湿 OR SU%=痹病 OR SU%=痹证 OR SU%=尪痹 OR SU%=RA) OR (TKA=类风湿关节炎 OR TKA=类风湿性关节炎 OR TKA=类风湿 OR TKA=痹病 OR TKA=痹证 OR TKA=尪痹 OR TKA=RA)) | 895 |

**Search Strategy in WanFang Database**

| **Steps** | **Search terms** | **Results** |
| --- | --- | --- |
| #1 | 全部:(青藤碱 or 青风藤碱 or 青风藤) and 全部:(类风湿关节炎 or 类风湿性关节炎 or 类风湿 or 痹病 or 痹证 or 尪痹 or RA) | 638 |

**Search Strategy in VIP Database**

| **Steps** | **Search terms** | **Results** |
| --- | --- | --- |
| #1 | (((任意字段=青藤碱 OR 任意字段=青风藤碱) OR 任意字段=青风藤) AND ((((((任意字段=类风湿关节炎 OR 任意字段=类风湿性关节炎) OR 任意字段=类风湿) OR 任意字段=痹病) OR 任意字段=痹证) OR 任意字段=尪痹) OR 任意字段=RA)) | 367 |

**Search Strategy in SinoMed Database**

| **Steps** | **Search terms** | **Results** |
| --- | --- | --- |
| #1 | "青藤碱"[不加权:扩展] | 658 |
| #2 | "青藤碱"[全部字段:智能] OR "青风藤碱"[全部字段:智能] OR "青风藤"[全部字段:智能] | 2546 |
| #3 | Search #1 OR #2 | 2546 |
| #4 | "关节炎, 类风湿"[不加权:扩展] | 171891 |
| #5 | "类风湿关节炎"[全部字段:智能] OR "类风湿性关节炎"[全部字段:智能] OR "类风湿"[全部字段:智能] OR "痹病"[全部字段:智能] OR "痹证"[全部字段:智能] OR "尪痹"[全部字段:智能] OR "RA"[全部字段:智能] | 706847 |
| #6 | Search #4 OR #5 | 728917 |
| #7 | Search #3 AND #6 | 498 |

**Table S1.** Composition and Reporting of Pharmaceutical Preparations in Included Studies

| Study ID | Metabolite Name | Manufacturer / Supplier | Batch / Lot Number | Purity | Authentication Method | Vehicle / Solvent |
| --- | --- | --- | --- | --- | --- | --- |
| Guo Wanyi et al. (2025) | Sinomenine | Hunan Zhengqing Pharmaceutical Group Co. Ltd. (Hunan, China) | NR | purity≥99% | HPLC | NR |
| Feng Yunjia et al. (2025) | Sinomenine | Xi’an Shennong Biotechnology Co. Ltd. | NR | purity≥98% | NR | NR |
| Liu Qingyang et al. (2024) | Sinomenine | MCE (MedChemExpress) | NR | NR | NR | normal saline |
| Li Juan et al. (2024) | Sinomenine | Solarbio Life Sciences, Beijing, China | NR | NR | NR | normal saline |
| Li Runze et al. (2023) | Sinomenine | NR | NR | NR | NR | normal saline |
| Li Juanmin et al. (2023) | Sinomenine | Hunan Zhengqing Pharmaceutical Group Co. Ltd. (Hunan, China) | NR | purity≥99% | HPLC | normal saline |
| Jiang Zhengmeng et al. (2023) | Sinomenine | NR | NR | NR | NR | NR |
| Jiang H et al. (2023) | Sinomenine | Selleck Chemicals (Shanghai, CHN) | S2359 | NR | NR | NR |
| Yi Lang et al. (2021) | Sinomenine | NR | NR | NR | NR | NR |
| Liao Kangsheng et al. (2021) | Sinomenine | ZhengQing company (Huaihua, Hunan, China) | NR | purity≥98% | HPLC | normal saline |
| Chen Liuzeng et al. (2021) | Sinomenine | NR | NR | NR | NR | NR |
| Xu Minmin et al. (2018) | Sinomenine | NR | NR | NR | NR | NR |
| Qian Xin et al. (2018) | Sinomenine | Hunan Zhengqing Pharmaceutical, Hunan, China | Z20010174 | NR | NR | normal saline |
| Liu Weiwei et al. (2018) | Sinomenine | Sigma-Aldrich Chemical Co. (St. Louis, MO, USA) | 115-53-7 | purity≥98% | HPLC | normal saline |
| Tong Bei et al. (2016) | Sinomenine | Nanjing Zelang Pharmaceutical Technology (Nanjing, China) | NR | purity＞98% | NR | NR |
| Tong Bei et al. (2015) | Sinomenine | Nanjing Zelang Pharmaceutical Technology Co., Ltd. (Nanjing, China) | NR | purity＞98% | NR | NR |
| Sun Yue et al. (2014) | Sinomenine | Zhengqing Pharmaceutical Group (Hunan, China) | NR | purity≥98% | HPLC | normal saline |
| Mu Hui et al. (2013) | Sinomenine | Zhengqing Pharmaceuticals Limited Co., Hunan, China | NR | NR | NR | normal saline |
| Zhou Hua et al. (2008) | Sinomenine | Shanxi Xuhuang Botanical Science & Technology Development Co., Ltd (Xi-an, China) | NR | purity≥98% | HPLC | normal saline |
| Xu Yuxiang et al. (2025) | Sinomenine | Shanghai Macklin Biochemical Technology Co., Ltd. | S794572 | NR | NR | normal saline |
| Feng Xia et al. (2025) | Sinomenine | Shanghai Macklin Biochemical Technology Co., Ltd. | S817690 | purity≥97% | NR | normal saline |
| Wen Wulong et al. (2024) | Sinomenine | Shaanxi Datian Biotechnology Co., Ltd. | DT0110522A | purity≥98% | NR | NR |
| Shang Wencui (2024) | Sinomenine | Shanghai Yuanye Biotechnology Co., Ltd. | NR | NR | NR | NR |
| Huang Yanping et al. (2023) | Sinomenine | Hunan Zhengqing Pharmaceutical Group Co. Ltd. (Hunan, China) | NR | NR | NR | NR |
| Zhang Junhao et al. (2022) | Sinomenine | Shaanxi Datian Biotechnology Co., Ltd. | DT0110522A | purity≥98% | NR | NR |
| Hu Mingyue et al. (2022) | Sinomenine | Hunan Zhengqing Pharmaceutical Group Co. Ltd. (Hunan, China) | NR | purity＞98% | NR | normal saline |
| Ji Jinyu (2021) | Sinomenine | NR | NR | NR | NR | Phosphate Buffered Saline |
| Zhao Jing et al. (2018) | Sinomenine | Baoji Yongjia Natural Plant Development Co., Ltd. | YJ0110822A | NR | NR | NR |
| Xu Wenjun (2018) | Sinomenine | Hunan Zhengqing Pharmaceutical Group Co. Ltd. (Hunan, China) | NR | NR | NR | normal saline |
| Zhou Haisong (2016) | Sinomenine | Dalian Meilun Biotechnology Co., Ltd. | NR | NR | NR | double-distilled water |
| Zhang Yuping (2016) | Sinomenine | Hunan Zhengqing Pharmaceutical Group Co. Ltd. (Hunan, China) | NR | NR | NR | normal saline |
| Gu Jie (2016) | Sinomenine | Xi'an Xiaocao Botanical Technology Co., Ltd. | 20131103 | NR | NR | distilled water |
| Xu Jia (2015) | Sinomenine | Hunan Zhengqing Pharmaceutical Group Co. Ltd. (Hunan, China) | 1107101 | NR | NR | normal saline |
| Zhang Mengying (2013) | Sinomenine | Hunan Zhengqing Pharmaceutical Group Co. Ltd. (Hunan, China) | 907101 | NR | NR | NR |
| Wang Shenzhi et al. (2012) | Sinomenine | Hunan Zhengqing Pharmaceutical Group Co. Ltd. (Hunan, China) | 20100625 | NR | NR | sterile water for injection |
| Ding Congzhu (2012) | Sinomenine | Hunan Zhengqing Pharmaceutical Group Co. Ltd. (Hunan, China) | 907101 | NR | NR | NR |
| Chen Xialin (2011) | Sinomenine | Hunan Zhengqing Pharmaceutical Group Co. Ltd. (Hunan, China) | 20100625 | NR | NR | sterile water for injection |
| Yang Desen et al. (2006) | Sinomenine | Baiyunshan Zhengqing Pharmaceutical Co., Ltd. | 20118 | NR | NR | 1% CMC-Na solution |

Note: In the included studies, the reporting of pharmaceutical preparation details varied. The manufacturer/supplier was reported in the majority of studies, with Hunan Zhengqing Pharmaceutical Group Co., Ltd. being the most frequently cited source of sinomenine. Batch/lot numbers were reported in a limited number of studies, while purity was reported in approximately half of the studies, typically ≥98% as determined by HPLC. Authentication methods were reported in a few studies (primarily HPLC), whereas the majority did not specify the method used. Vehicle/solvent information was reported in approximately half of the studies, with normal saline being the most commonly used. “NR” indicates that the information was not reported in the original study.

**Table S2.** Safety Profile of Sinomenine in Included Studies

| Study ID | Reported Findings |
| --- | --- |
| Guo Wanyi et al. (2025) | The clinical application of sinomenine is reported to be limited by the need for relatively high doses to achieve therapeutic effects, which may increase the risk of allergic reactions in some patients. |
| Feng Yunjia et al. (2025) | NR |
| Liu Qingyang et al. (2024) | Body weight: In the sinomenine treatment group, mouse body weight showed no obvious decrease compared with the control group (Fig. 9A). Histopathology: H&E staining of liver and kidney tissues showed no obvious inflammation or histopathological changes in the sinomenine treatment group (Fig. 9D). No obvious toxicity was observed in the high-dose group (100 mg/kg). |
| Li Juan et al. (2024) | Body weight: In the sinomenine treatment groups (25, 50 mg/kg), rat body weight was increased compared with the rheumatoid arthritis model group (Fig. 2B), with no body weight loss or other signs of toxicity. Immune organ index: Sinomenine reduced the elevated spleen index and thymus index in rats with rheumatoid arthritis (Fig. 3C, 3D), reflecting immunomodulatory effects rather than toxicity. |
| Li Runze et al. (2023) | NR |
| Li Juanmin et al. (2023) | Body weight: In the CIA mouse model, body weight in the high-dose sinomenine group (100 mg/kg) was improved compared with the model group (Fig. 8c), with no body weight loss or other signs of toxicity. |
| Jiang Zhengmeng et al. (2023) | Body weight: In the sinomenine treatment groups (30, 120, 240 mg/kg), rat body weight was increased compared with the model group (Fig. 1a), with no body weight loss or other signs of toxicity. Immune organ index: Spleen index and thymus index in the sinomenine treatment groups were decreased compared with the model group (Fig. 1i, 1j), reflecting immunomodulatory effects rather than toxicity. |
| Jiang H et al. (2023) | Body weight: In the sinomenine treatment group, mouse body weight showed no significant difference compared with the model group (Fig. 1E), with no body weight loss or other signs of toxicity. |
| Yi Lang et al. (2021) | NR |
| Liao Kangsheng et al. (2021) | NR |
| Chen Liuzeng et al. (2021) | NR |
| Xu Minmin et al. (2018) | NR |
| Qian Xin et al. (2018) | Bone marrow suppression (complete blood count): White blood cells, red blood cells, hemoglobin, and platelet levels in the sinomenine treatment group showed no significant difference compared with the control group (Table IX), indicating that sinomenine did not induce bone marrow suppression. |
| Liu Weiwei et al. (2018) | NR |
| Tong Bei et al. (2016) | NR |
| Tong Bei et al. (2015) | NR |
| Sun Yue et al. (2014) | NR |
| Mu Hui et al. (2013) | NR |
| Zhou Hua et al. (2008) | NR |
| Xu Yuxiang et al. (2025) | NR |
| Feng Xia et al. (2025) | Kidney function indicators: Serum creatinine, urea, and uric acid levels in the sinomenine treatment group were within the normal range (Fig. 3B). Liver and kidney histopathology: H&E staining showed that in the sinomenine treatment group, hepatocytes were arranged in an orderly manner with intact structure, and renal tubular epithelial cells were arranged in an orderly manner with normal glomerular structure; no abnormal proliferation or inflammatory cell infiltration was observed (Fig. 3C, 3D). |
| Wen Wulong et al. (2024) | NR |
| Shang Wencui (2024) | H&E staining showed no obvious tissue damage in major organs (heart, liver, spleen, lung, kidney) in the sinomenine group (Fig. 3-12). |
| Huang Yanping et al. (2023) | NR |
| Zhang Junhao et al. (2022) | NR |
| Hu Mingyue et al. (2022) | Heart, liver, spleen, lung, kidney, thymus, and adrenal gland indices in the sinomenine hydrochloride treatment group showed no significant difference compared with the control group (Fig. 2), indicating no obvious toxic effects of sinomenine hydrochloride on major organs and tissues. |
| Ji Jinyu (2021) | NR |
| Zhao Jing et al. (2018) | In the discussion section, it is noted that in the high-dose sinomenine group (120 mg/kg), joint histopathology scores and serum IL-1, IL-6, and IL-10 levels showed no significant differences compared with the model group, which may suggest that the immunosuppressive pharmacological effects of sinomenine are reduced at high doses, potentially leading to adverse effects on organs. A cited reference [16] indicates that "high-dose sinomenine can cause liver injury in rats, and the degree of organ injury is positively correlated with the concentration of the drug in the tissues." |
| Xu Wenjun (2018) | NR |
| Zhou Haisong (2016) | In the sinomenine treatment groups (40, 80, 120 mg/kg), thymus index, spleen index, liver index, and kidney index showed no significant differences compared with the model group (Table 2-3, Fig. 2-5), except for a decrease in thymus index in certain dose groups, which may be related to immunomodulatory effects. No obvious abnormalities in the appearance or color of organs were observed. Body weight: No significant differences in body weight were observed between the sinomenine treatment groups and the model group (Table 2-2, Fig. 2-3, 2-4), except that body weight in the 120 mg/kg group was greater than that of the model group on day 9. |
| Zhang Yuping (2016) | NR |
| Gu Jie (2016) | NR |
| Xu Jia (2015) | NR |
| Zhang Mengying (2013) | Platelet count (PLT) and hemoglobin (Hb) levels in the medium-dose sinomenine group showed no significant differences compared with the model group (Table 2-1, Fig. 2-8, 2-9). |
| Wang Shenzhi et al. (2012) | NR |
| Ding Congzhu (2012) | NR |
| Chen Xialin (2011) | NR |
| Yang Desen et al. (2006) | NR |

**Note:** NR, not reported. Body weight was reported in 6 studies (Liu Qingyang et al., 2024; Li Juan et al., 2024; Li Juanmin et al., 2023; Jiang Zhengmeng et al., 2023; Jiang H et al., 2023; Zhou Haisong, 2016), major organ histopathology in 3 studies (Liu Qingyang et al., 2024; Feng Xia et al., 2025; Shang Wencui, 2024), immune organ indices in 4 studies (Li Juan et al., 2024; Jiang Zhengmeng et al., 2023; Hu Mingyue et al., 2022; Zhou Haisong, 2016), and myelosuppression parameters in 2 studies (Qian Xin et al., 2018; Zhang Mengying, 2013).

**Table S3.** Evidence Grading for the Mechanisms of Sinomenine in RA

|  | Pathway/Mechanism | Supporting Literature | Evidence Level | Rationale for Grading |
| --- | --- | --- | --- | --- |
| **Antioxidant Stress** | SIN → Activates Nrf2 → Upregulates HO-1/NQO1 | Li Juan et al. (2024), Liao Kangsheng et al. (2021), Zhou Haisong (2016) | A (Well-established) | This classic antioxidant pathway is confirmed by at least three independent studies using both in vivo and in vitro models. |
|  | SIN → Increases SOD/CAT/GPx/GSH | Li Juan et al. (2024), Xu Minmin et al. (2018), Xu Yuxiang et al. (2025) | A (Well-established) | The upregulation of these key antioxidant enzymes is a consistent finding across multiple studies, providing robust evidence. |
|  | SIN → Decreases ROS/MDA | Feng Yunjia et al. (2025), Li Juanmin et al. (2023), Shang Wencui (2024), Xu Minmin et al. (2018), Xu Yuxiang et al. (2025) | A (Well-established) | As a direct measure of antioxidant efficacy, the reduction of ROS/MDA is one of the most frequently reported and well-established effects. |
|  | SIN → Oxidative Stress → Indirectly inhibits NLRP3 | Guo Wanyi et al. (2025) | C (Speculative) | This indirect link is proposed by a single study. While biologically plausible (ROS activates NLRP3), this specific crosstalk for SIN needs more direct experimental validation. |
|  | SIN → Oxidative Stress → Indirectly inhibits Osteoclastogenesis | Guo Wanyi et al. (2025) | C (Speculative) | Similar to the above, this is a reasonable hypothesis from a single paper that requires further dedicated studies to establish a direct causal link. |
| **Anti-inflammatory Effects** | SIN → Inhibits NF-κB pathway, reducing inflammatory factors | Feng Yunjia et al. (2025), Li Juan et al. (2024), Jiang Zhengmeng et al. (2023), Jiang H et al. (2023), Yi Lang et al. (2021), Xu Minmin et al. (2018), Feng Xia et al. (2025), Shang Wencui (2024), Ji Jinyu (2021), Zhang Yuping (2016) | A (Well-established) | This is the most extensively documented mechanism, supported by a large body of literature, making it a cornerstone of SIN’s anti-inflammatory action. |
|  | SIN → NF-κB inhibition → Indirectly affects NLRP3 | Xu Yuxiang et al. (2025) | C (Speculative) | This crosstalk is suggested by a single paper. Given NF-κB is a priming signal for NLRP3, the link is logical but requires more focused research to be considered established. |
|  | SIN → Inhibits NLRP3 inflammasome (and downstream effectors) | Li Juanmin et al. (2023), Xu Yuxiang et al. (2025), Huang Yanping et al. (2023) | B (Strong) | Supported by three independent studies providing direct evidence for the inhibition of the inflammasome complex. This constitutes strong, but not yet consensus, evidence. |
|  | SIN → Inhibits MAPK pathway (p38), iNOS/COX-2 ↓, NO/PGE2 ↓ | Feng Yunjia et al. (2025), Li Juanmin et al. (2023); Jiang Zhengmeng et al. (2023), Jiang H et al. (2023), Xu Minmin et al. (2018) | A (Well-established) | With support from five independent papers, the inhibition of the MAPK/p38 axis by SIN is a well-established mechanism. |
|  | SIN → Inhibits JAK/STAT pathway, reducing inflammatory factors | Xu Wenjun (2018) | C (Speculative) | This pathway is supported by a single study. It represents a promising but preliminary finding that needs independent verification. |
|  | SIN → Inhibits PI3K/Akt pathway, affecting proliferation | Liu Qingyang et al. (2024) | C (Speculative) | This entire mechanism is detailed within a single comprehensive study. While internally consistent, it awaits confirmation from other research groups. |
|  | SIN → Inhibits PI3K/Akt pathway, reducing inflammatory factors | Liu Qingyang et al. (2024) | C (Speculative) | This entire mechanism is detailed within a single comprehensive study. While internally consistent, it awaits confirmation from other research groups. |
|  | SIN → Reduced inflammatory factors → Indirectly affects RANKL | Sun Yue et al. (2014), Zhou Haisong (2016) | B (Strong) | The link between pro-inflammatory cytokines (like TNF-α) and RANKL expression is a known concept in RA pathogenesis. These papers support the two ends of this pathway, making the indirect link strongly plausible. |
| **Immunoregulation** | SIN → Modulates Th17/Treg balance (Th17↓, Treg↑) | Liu Qingyang et al. (2024), Jiang Zhengmeng et al. (2023), Tong Bei et al. (2016), Tong Bei et al. (2015) | A (Well-established) | This effect is confirmed by multiple high-quality studies, including mechanistic explorations (e.g., via AhR), establishing it as a key immunoregulatory mechanism. |
|  | SIN → Modulates gut immunity (activates AhR → upregulates CYP1A1) | Jiang Zhengmeng et al. (2023), Qian Xin et al. (2018) (预测), Tong Bei et al. (2016) | B (Strong) | This emerging mechanism is supported by strong evidence from mechanistic studies (e.g., FMT, AhR inhibitors), though it is a more recent area of focus. |
|  | SIN → Inhibits NETosis (reducing PAD4, CitH3, NE) | Jiang H et al. (2023) | C (Speculative) | This novel mechanism is detailed in a single paper. It is a highly relevant but preliminary finding that requires further validation. |
|  | SIN → Modulates Macrophage/Monocyte populations | Feng Yunjia et al. (2025), Liu Weiwei et al. (2018), Shang Wencui (2024) | B (Strong) | Multiple studies confirm SIN’s ability to modulate macrophage polarization or reduce inflammatory monocyte populations, providing strong evidence for this role. |
|  | AhR directly affects Th17/Treg balance | Jiang Zhengmeng et al. (2023), Tong Bei et al. (2016) | A (Well-established) | These key papers use mechanistic tools (AhR inhibitors/siRNA) to directly prove that AhR activation by SIN is responsible for modulating the Th17/Treg balance. |
|  | Modulated Th17/Treg balance → Indirectly affects inflammatory factors | Liu Qingyang et al. (2024), Jiang Zhengmeng et al. (2023), Tong Bei et al. (2016), Tong Bei et al. (2015) | A (Well-established) | This is a fundamental immunological principle (Th17 produces pro-inflammatory cytokines, Treg produces anti-inflammatory ones), and these studies confirm this functional outcome in the context of SIN treatment. |
| **Protection Against Joint Destruction** | SIN → Modulates RANKL/OPG balance (RANKL↓, OPG↑) | Li Juan et al. (2024), Sun Yue et al. (2014), Xu Jia (2015), Zhang Mengying (2013), Ding Congzhu (2012) | A (Well-established) | A large number of studies consistently report this effect on the key bone metabolism axis, making it a well-established mechanism of bone protection. |
|  | SIN → Inhibits Osteoclastogenesis, CTSK/MMP-9/TRAP/Oscar ↓ | Guo Wanyi et al. (2025), Ji Jinyu (2021), Xu Jia (2015), Ding Congzhu (2012) | A (Well-established) | The functional consequence of RANKL/OPG modulation is directly confirmed by multiple studies showing reduced osteoclast formation and activity markers. |
|  | SIN → Inhibits MMPs (MMP-2, MMP-9, etc.) | Guo Wanyi et al. (2025), Li Juan et al. (2024), Xu Minmin et al. (2018), Zhou Hua et al. (2008), Gu Jie (2016) | A (Well-established) | The inhibition of these cartilage-degrading enzymes is a consistent finding across many independent studies. |
|  | SIN → Upregulates TIMPs | Li Juan et al. (2024), Zhou Hua et al. (2008) | B (Strong) | Supported by two independent studies, the upregulation of endogenous MMP inhibitors (TIMPs) constitutes strong evidence for an additional protective mechanism. |

**Table S4.** Data Extracted for GRIM Testing: Integer Outcomes in Included

| Study ID | Outcome Indicator | Sample Size (n)-SIN | Reported Mean-SIN | Consistency Check-SIN | Sample Size (n)-MOD | Reported Mean-MOD | Consistency Check-MOD | One-by-One Sensitivity Analysis |
| --- | --- | --- | --- | --- | --- | --- | --- | --- |
| Qian Xin et al. (2018) | Arthritis index | 10 | 6.4 | 64 | 10 | 13.5 | 135 | Consistent |
|  | Arthritis index | 10 | 10.5 | 105 | 10 | 13.5 | 135 | Consistent |
|  | Arthritis index | 10 | 10.5 | 105 | 10 | 13.5 | 135 | Consistent |
| Xu Yuxiang et al. (2025)* | Arthritis index | 10 | 1.12 | 11.2 | 10 | 8.61 | 86.1 | -2.8190243 ~-2.0446832 |
|  | Arthritis index | 10 | 4.67 | 46.7 | 10 | 8.61 | 86.1 | -2.8834565 ~-2.088412 |
|  | Arthritis index | 10 | 7.02 | 70.2 | 10 | 8.61 | 86.1 | -2.9291384 ~-2.1254263 |
| Wen Wulong et al. (2024)* | Arthritis index | 8 | 2.33 | 18.64 | 8 | 3.69 | 29.52 | -2.8607876 ~-2.0725205 |
| Zhang Junhao et al. (2022)* | Arthritis index | 5 | 2.32 | 11.6 | 5 | 3.65 | 18.25 | -2.8768759 ~-2.0849679 |
|  | Arthritis index | 5 | 1.87 | 9.35 | 5 | 3.65 | 18.25 | -2.8685913 ~-2.0788965 |
| Hu Mingyue et al. (2022) | Arthritis index | 5 | 2.4 | 12 | 5 | 6.8 | 34 | Consistent |
| Xu Wenjun (2018)* | Arthritis index | 7 | 2.29 | 16.03 | 6 | 9.83 | 58.98 | -2.8425972 ~-2.0606112 |
| Zhou Haisong (2016)* | Arthritis index | 8 | 10.63 | 85.04 | 8 | 17.22 | 137.76 | -2.9145017 ~-2.1130385 |
|  | Arthritis index | 8 | 10 | 80 | 8 | 17.22 | 137.76 | -2.899771 ~-2.1010928 |
|  | Arthritis index | 8 | 12.67 | 101.36 | 8 | 17.22 | 137.76 | -2.9270911 ~-2.1251767 |
| Zhang Yuping (2016)* | Arthritis index | 8 | 1.67 | 13.36 | 9 | 3.33 | 29.97 | -2.9180205 ~-2.1157641 |
| Wang Shenzhi et al. (2012) | Arthritis index | 10 | 5.8 | 58 | 10 | 8.6 | 86 | Consistent |
| Chen Xialin (2011) | Arthritis index | 10 | 5.9 | 59 | 10 | 8.6 | 86 | Consistent |
| Yang Desen et al. (2006)* | Arthritis index | 12 | 4.1 | 49.2 | 12 | 8.7 | 104.4 | -2.8596702 ~-2.0719566 |
|  | Arthritis index | 12 | 4.7 | 56.4 | 12 | 8.7 | 104.4 | -2.8725207 ~-2.0806527 |
|  | Arthritis index | 12 | 5.1 | 61.2 | 12 | 8.7 | 104.4 | -2.8819892 ~-2.0871127 |
| Qian Xin et al. (2018) | Histological scores | 8 | 3.75 | 30 | 8 | 5.50 | 44 | Consistent |
|  | Histological scores | 8 | 4.00 | 32 | 8 | 5.50 | 44 | Consistent |
|  | Histological scores | 8 | 4.25 | 34 | 8 | 5.50 | 44 | Consistent |
| Hu Mingyue et al. (2022) | Histological scores | 5 | 2.40 | 12 | 5 | 5.20 | 26 | Consistent |
| Wang Shenzhi et al. (2012) | Histological scores | 10 | 1.90 | 19 | 10 | 3.70 | 37 | Consistent |
| Chen Xialin (2011) | Histological scores | 10 | 1.90 | 19 | 10 | 3.70 | 37 | Consistent |
| Excluding All 7 Suspect Studies | | | | | | | | -2.53 ~-1.71 |

Note: SIN, sinomenine; MOD, model. The seven GRIM-inconsistent studies are marked in red and with asterisks.

**Table S5.** Visual inspection record of figures in included studies.

| Study ID | Figures examined | Duplication? | Splicing? | Other obvious anomaly? | Inspection completed by (initials) |
| --- | --- | --- | --- | --- | --- |
| Guo Wanyi et al. (2025) | Fig. 4B, D, H | No | No | No | LG/Z-YC |
| Feng Yunjia et al. (2025) | Fig. 7E; 8E | No | No | No | LG/Z-YC |
| Liu Qingyang et al. (2024) | Fig. 8C; 9B, C; 10A, B,C | No | No | No | LG/Z-YC |
| Li Juan et al. (2024) | Fig. 5C, D, E, G; 9A, B, E | No | No | No | LG/Z-YC |
| Li Runze et al. (2023) | Fig. 5A, B, E, F, G | No | No | No | LG/Z-YC |
| Li Juanmin et al. (2023) | Fig. 8E; 9D, E, G | No | No | No | LG/Z-YC |
| Jiang Zhengmeng et al. (2023) | Fig. 1B, C, F, K, L | No | No | No | LG/Z-YC |
| Jiang H et al. (2023) | Fig. 1D; 3A, C, D | No | No | No | LG/Z-YC |
| Yi Lang et al. (2021) | Fig. 1A, B, D | No | No | No | LG/Z-YC |
| Liao Kangsheng et al. (2021) | Fig. 1A, E, G, H, J | No | No | No | LG/Z-YC |
| Chen Liuzeng et al. (2021) | Fig. 5B;7A, B, C | No | No | No | LG/Z-YC |
| Xu Minmin et al. (2018) | Fig. 2;4A, B, C;7B | No | No | No | LG/Z-YC |
| Liu Weiwei et al. (2018) | Fig. 4B; 5A, E, F, I | No | No | No | LG/Z-YC |
| Tong Bei et al. (2016) | Fig. 6A, F, G | No | No | No | LG/Z-YC |
| Tong Bei et al. (2015) | Fig. 1A, D; 2A, B; 6B, C | No | No | No | LG/Z-YC |
| Sun Yue et al. (2014) | Fig. 1A, B; 3A | No | No | No | LG/Z-YC |
| Mu Hui et al. (2013) | Fig. 4B, C, D;6A | No | No | No | LG/Z-YC |
| Zhou Hua et al. (2008) | Fig. 1B, C; 2A, B; 3D | No | No | No | LG/Z-YC |
| Feng Xia et al. (2025) | Fig. 1B, C, D; 3A | No | No | No | LG/Z-YC |
| Shang Wencui (2024) | Fig. 3-6C; 3-8 | No | No | No | LG/Z-YC |
| Huang Yanping et al. (2023) | Fig. 1A, B; 3D | No | No | No | LG/Z-YC |
| Hu Mingyue et al. (2022) | Fig. 1; 3 | No | No | No | LG/Z-YC |
| Ji Jinyu (2021) | Fig. 1; 6B | No | No | No | LG/Z-YC |
| Zhao Jing et al. (2018) | Fig. 2 | No | No | No | LG/Z-YC |
| Gu Jie (2016) | Fig. 4; 5; 9; 10; 11 | No | No | No | LG/Z-YC |
| Xu Jia (2015) | Fig. 3-4 | No | No | No | LG/Z-YC |
| Zhang Mengying (2013) | Fig. 2-5 | No | No | No | LG/Z-YC |
| Ding Congzhu (2012) | Fig. 1-3 | No | No | No | LG/Z-YC |

**Fig S1. Subgroup analysis of arthritis index by dose.**

**
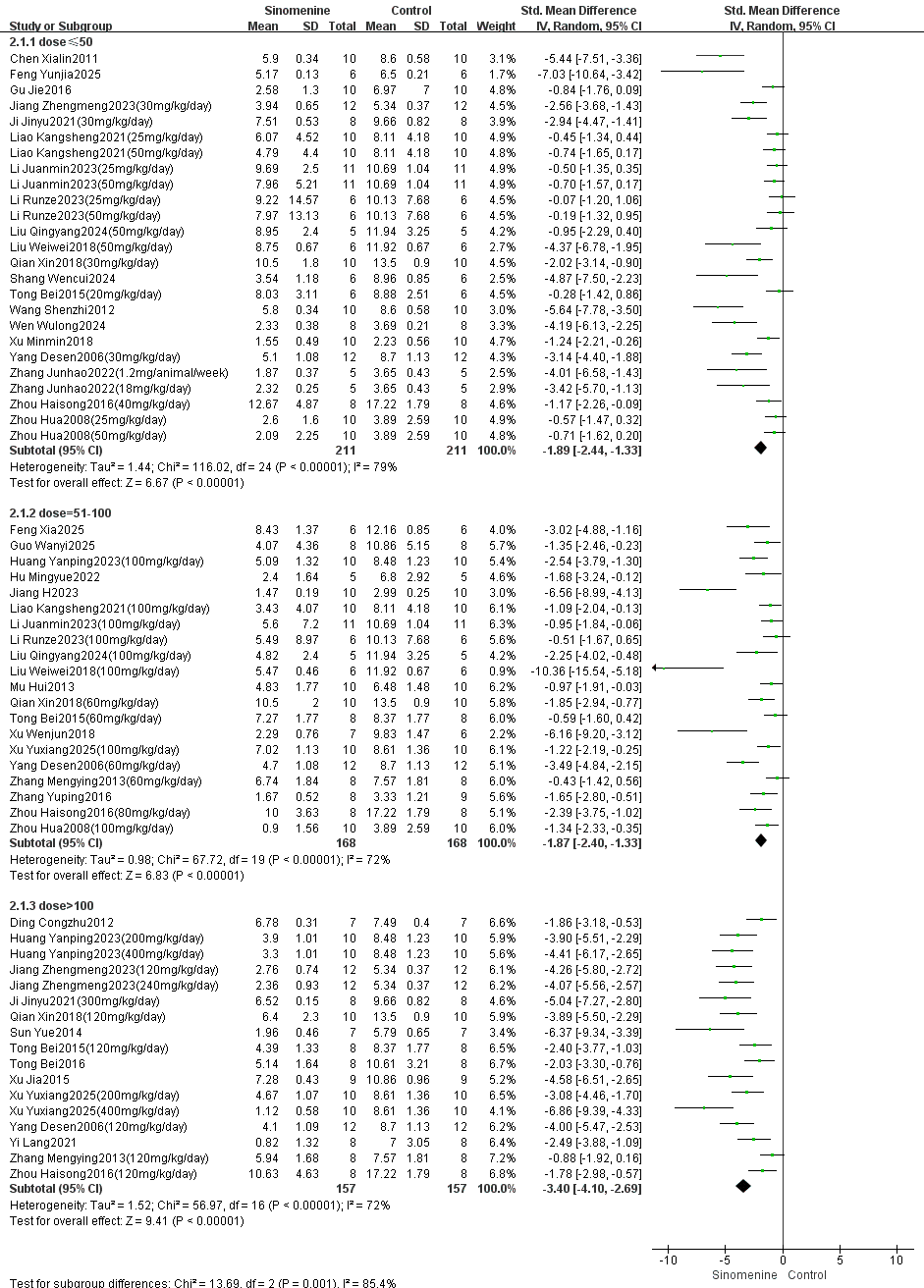
**

**Fig S2. Subgroup analysis of arthritis index by intervention duration.**

**
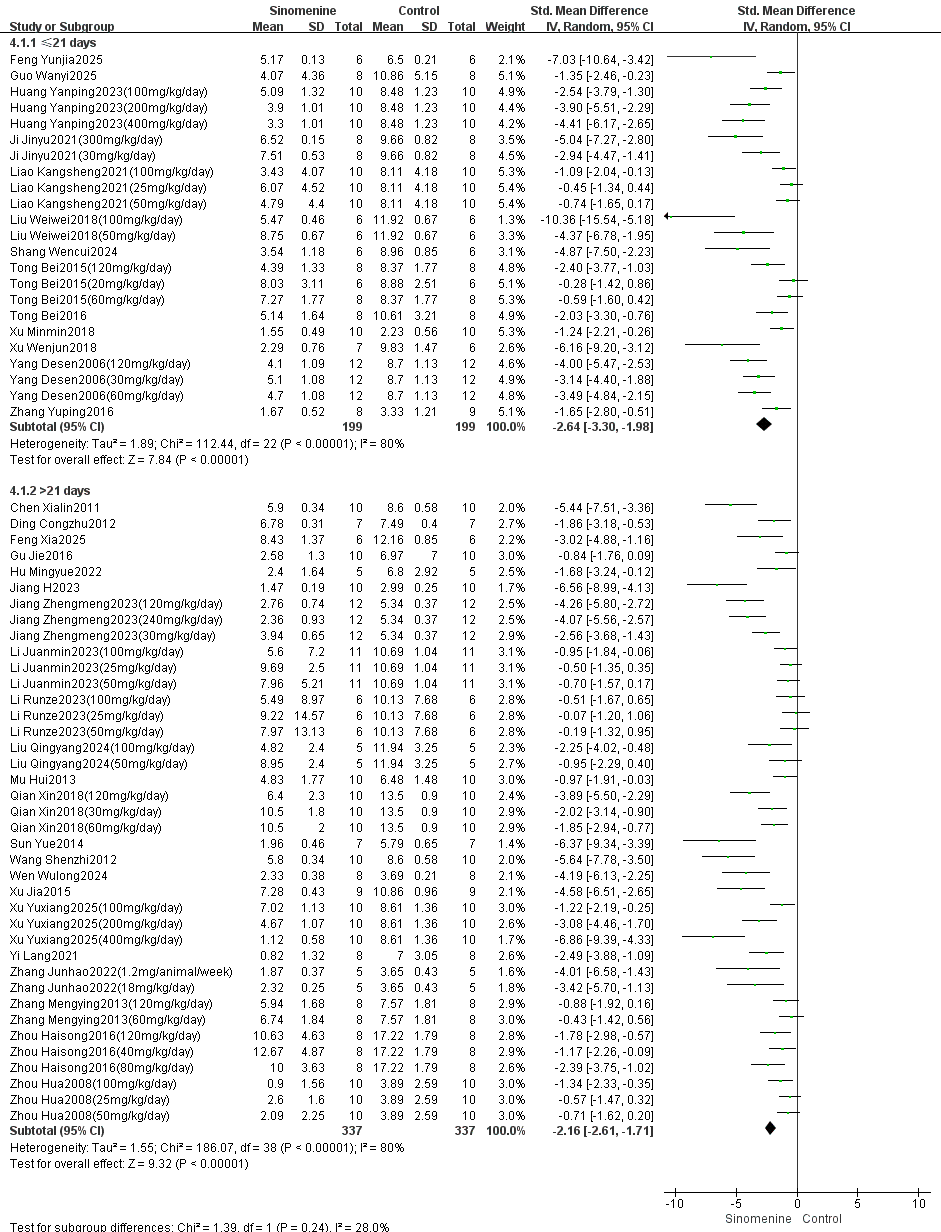
**

**Fig S3. Subgroup analysis of arthritis index by administration route.**

**
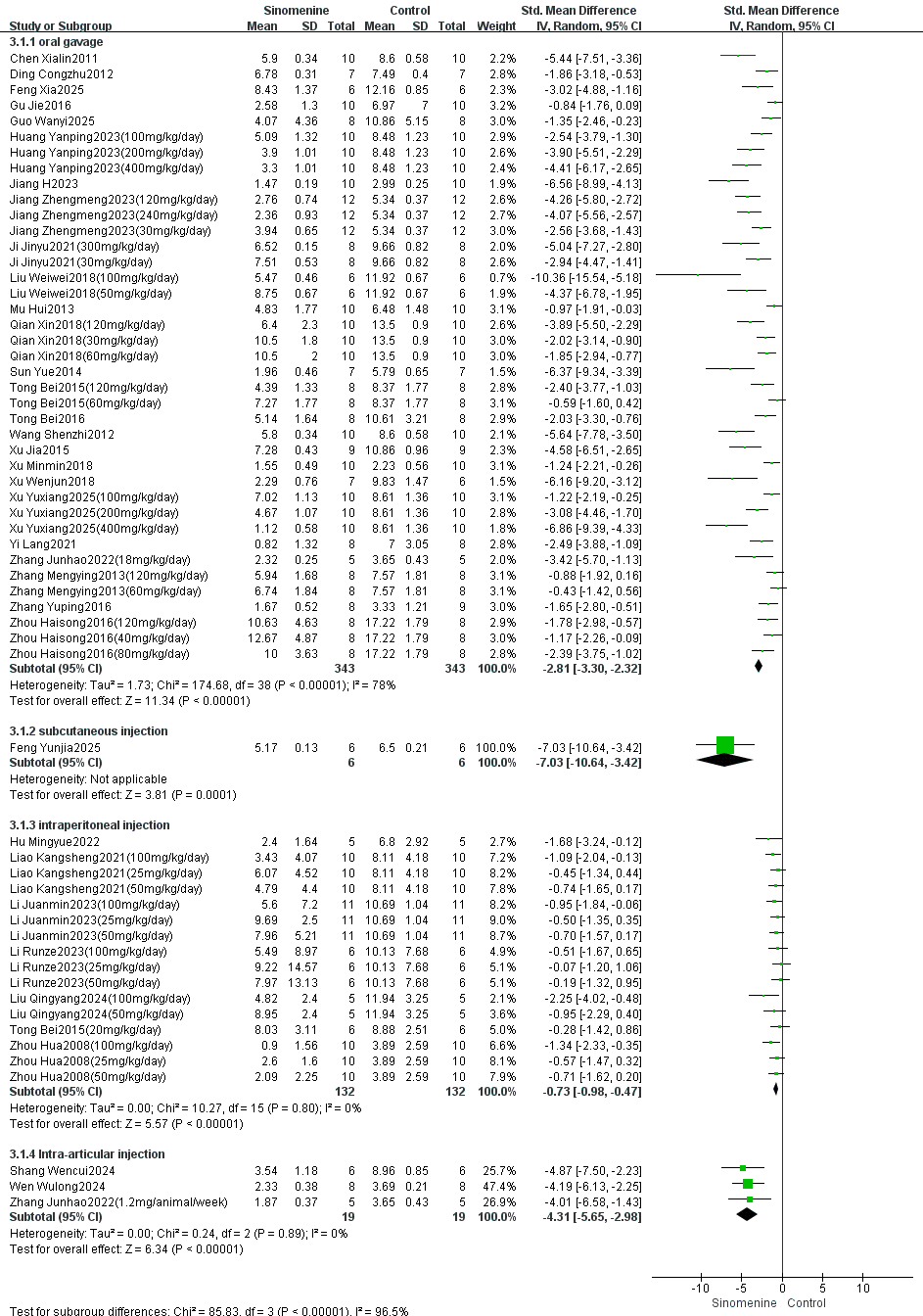
**

**Fig S4. Subgroup analysis of paw volume by dose.**

**
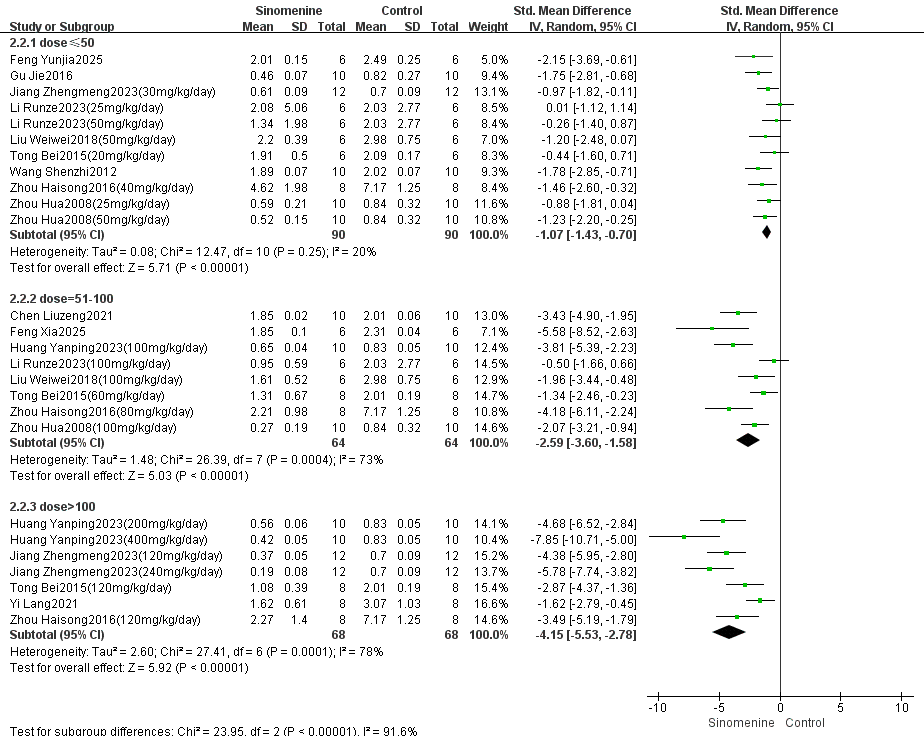
**

**Fig S5. Subgroup analysis of paw volume by intervention duration.**

**
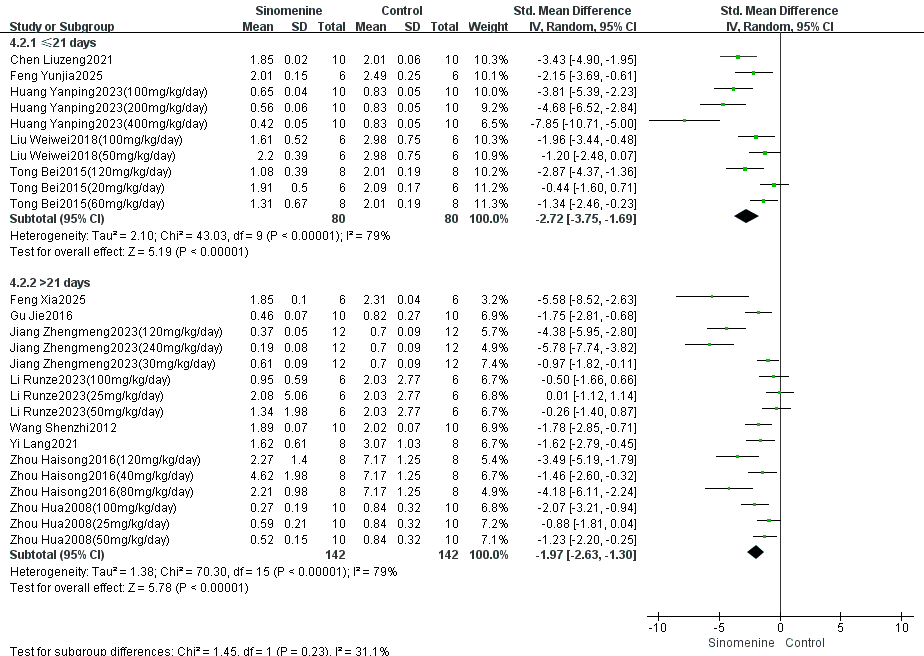
**

**Fig S6. Subgroup analysis of paw volume by administration route.**

**
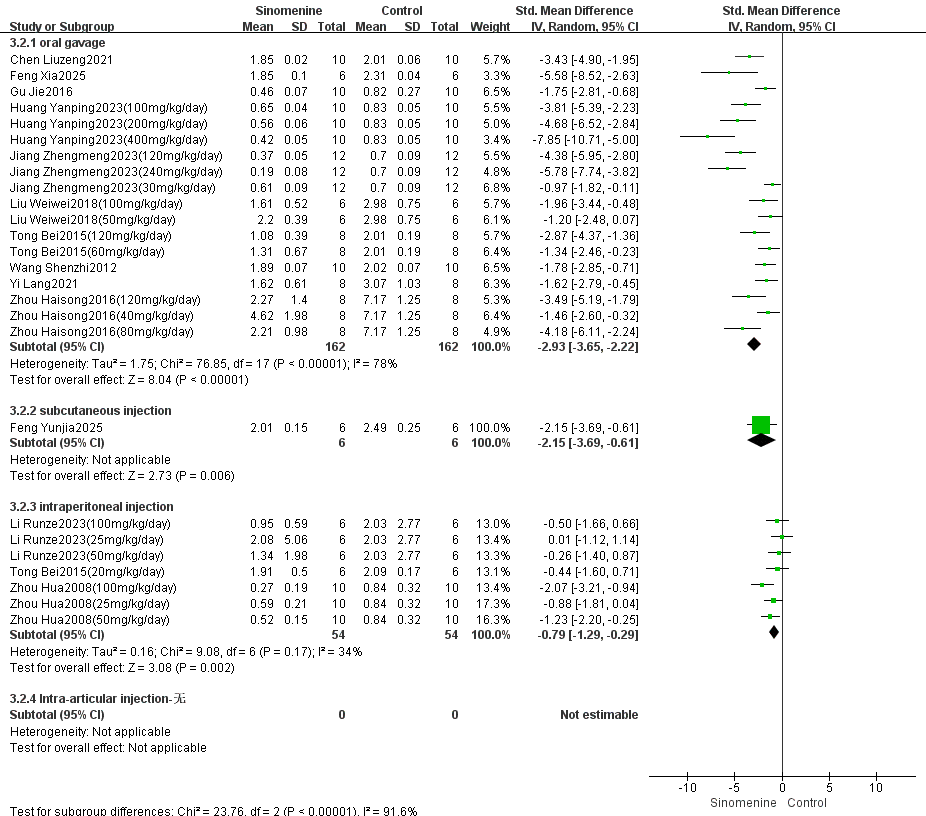
**

**Fig S7. Subgroup analysis of TNF-α by dose.**

**
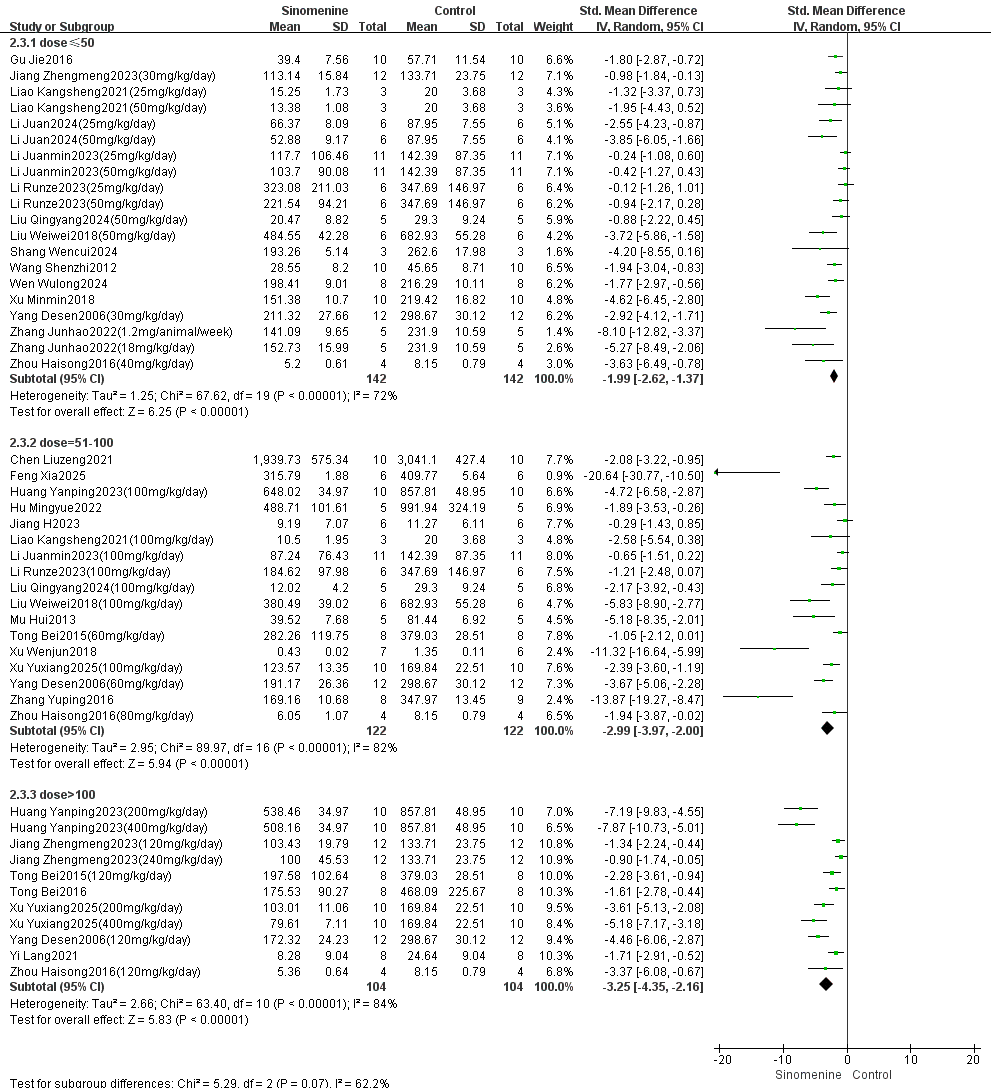
**

**Fig S8. Subgroup analysis of TNF-α by intervention duration.**

**
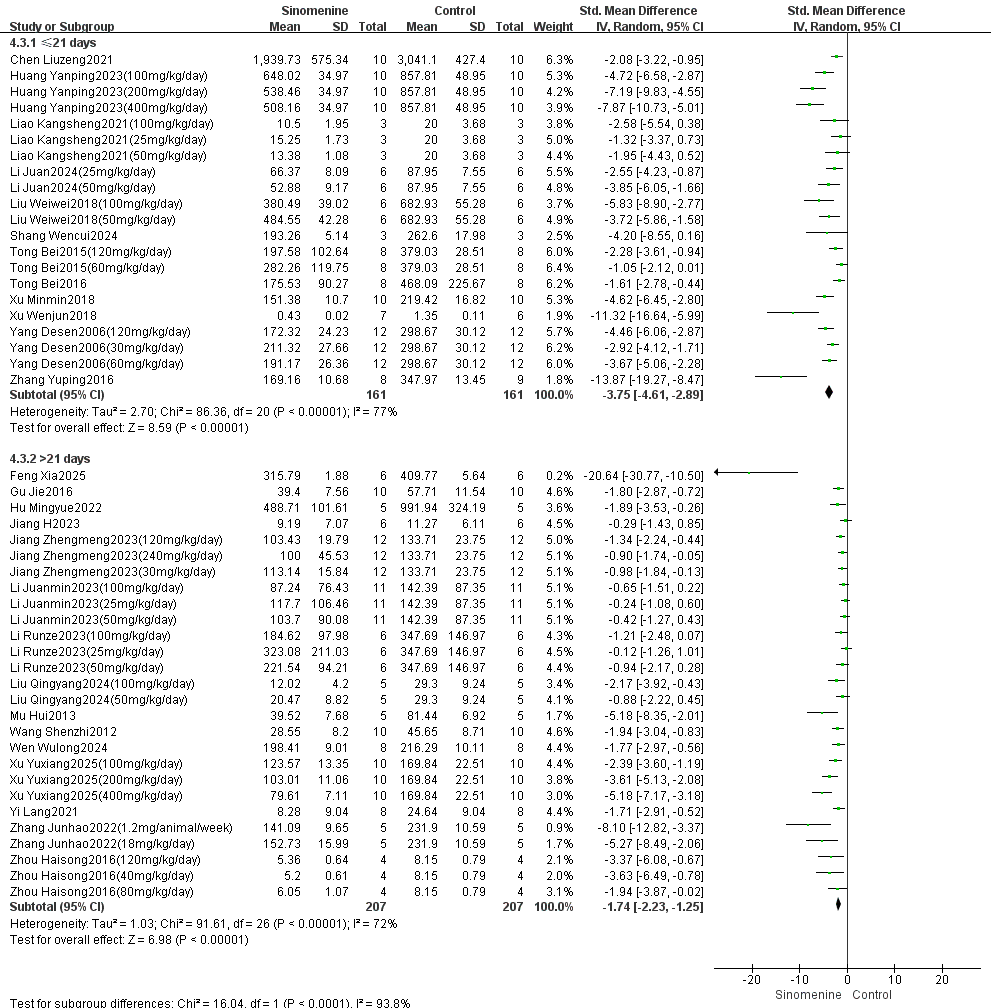
**

**Fig S9. Subgroup analysis of TNF-α by administration route.**

**
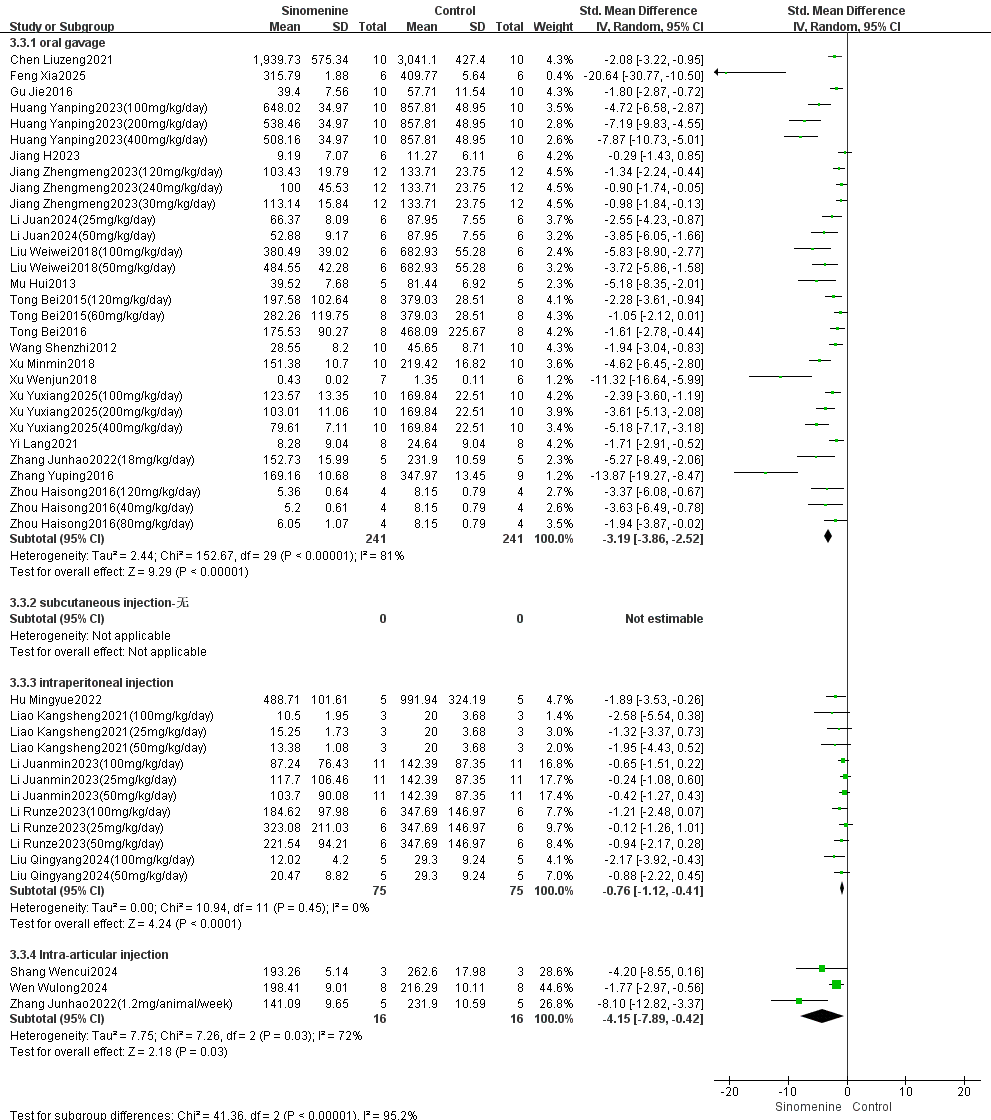
**

**Fig S10. Subgroup analysis of IL-1β by dose.**

**
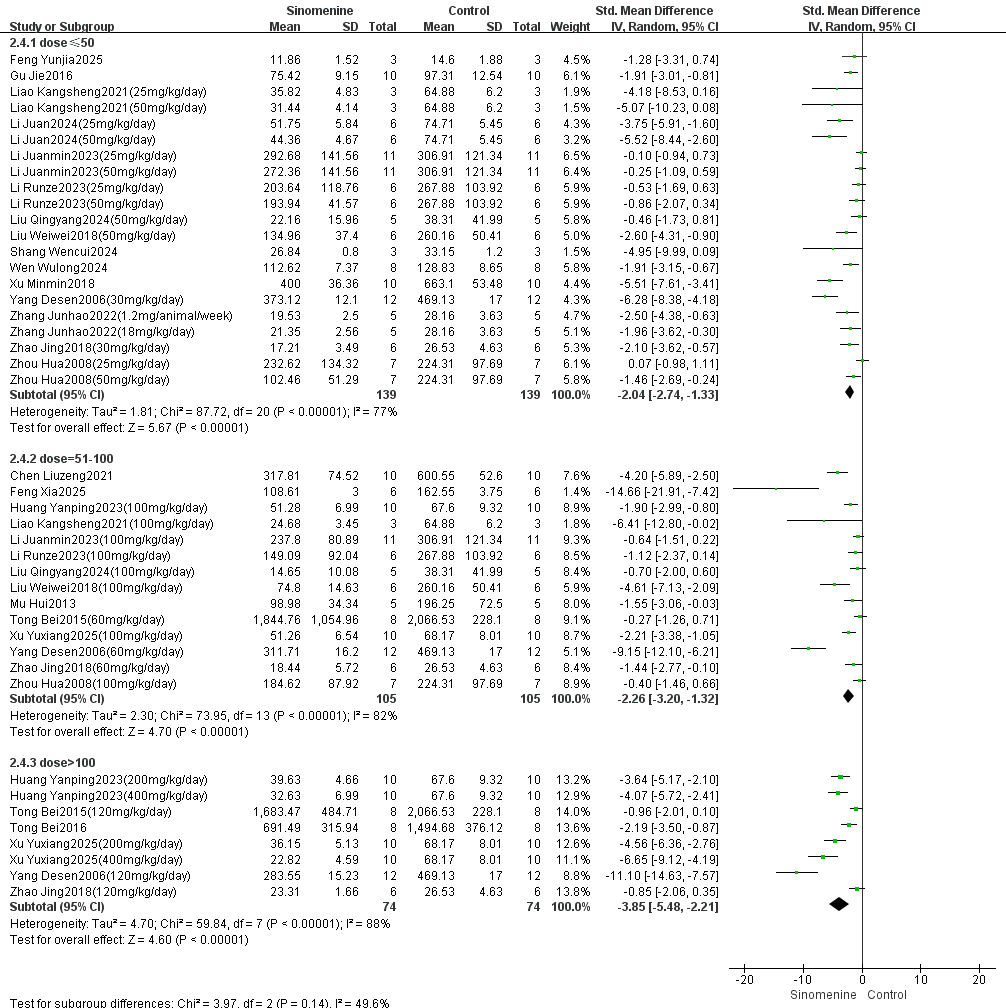
**

**Fig S11. Subgroup analysis of IL-1β by intervention duration.**

**
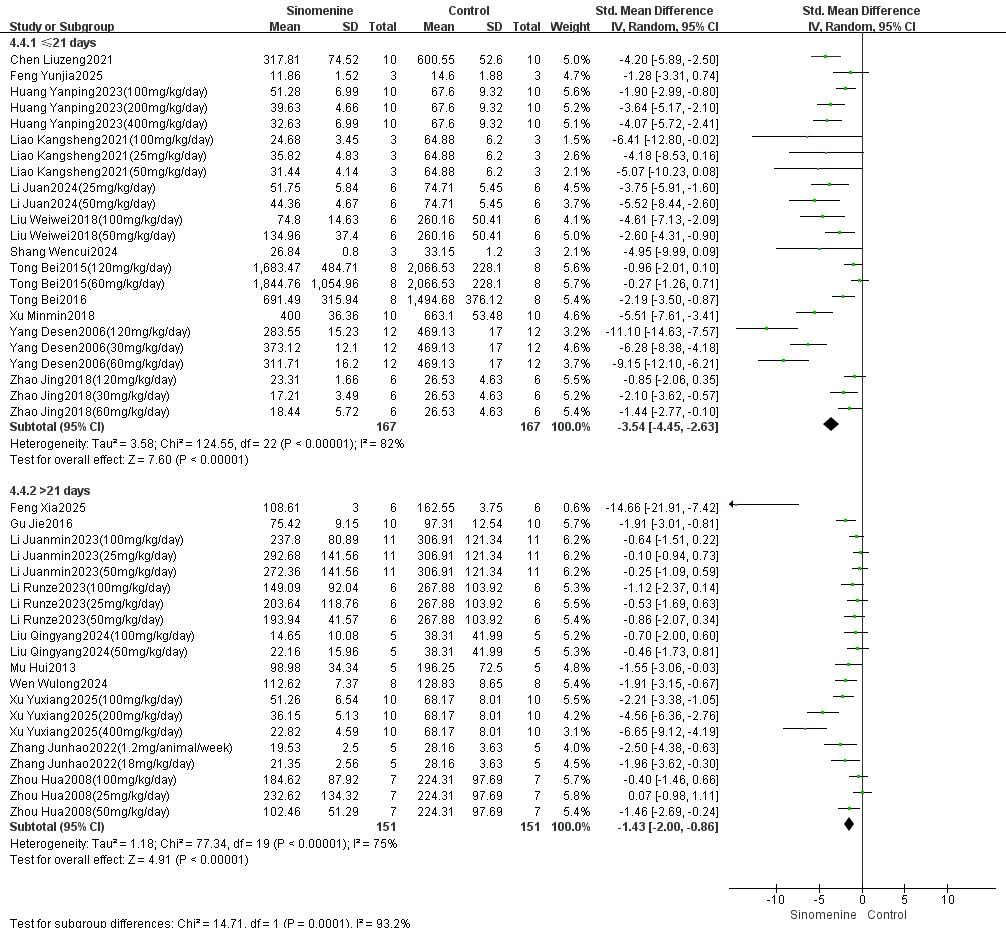
**

**Fig S12. Subgroup analysis of IL-1β by administration route.**

**
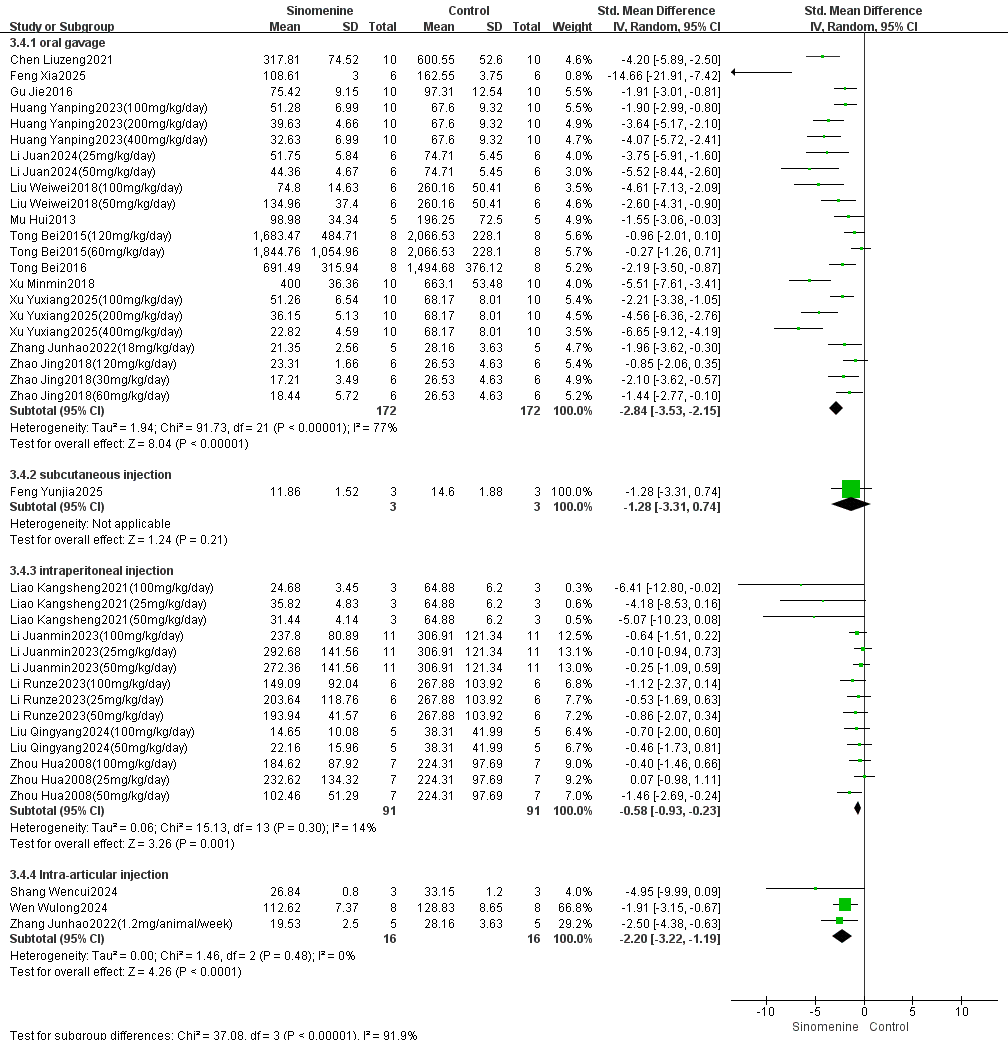
**

**Fig S13. Subgroup analysis of IL-6 by dose.**

**
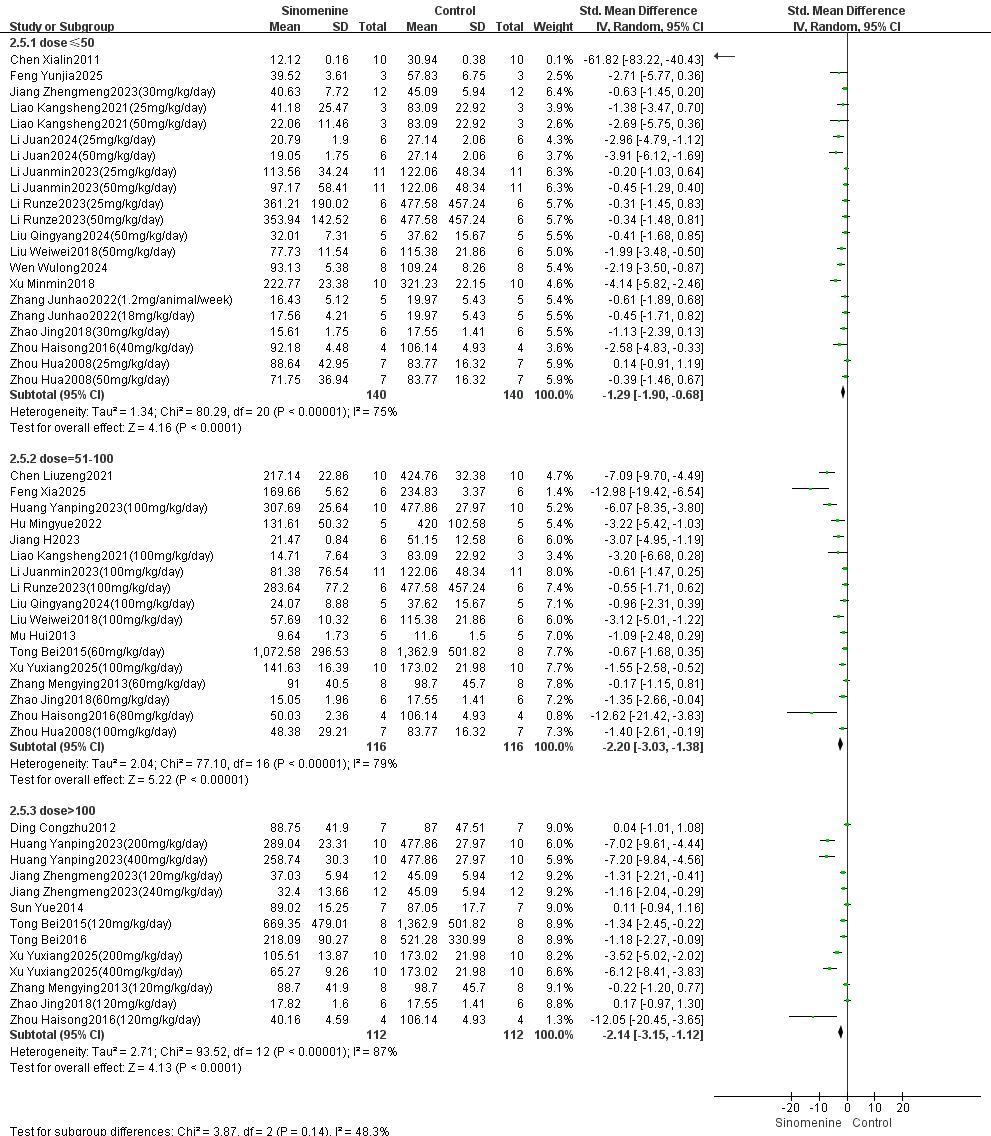
**

**Fig S14. Subgroup analysis of IL-6 by intervention duration.**

**
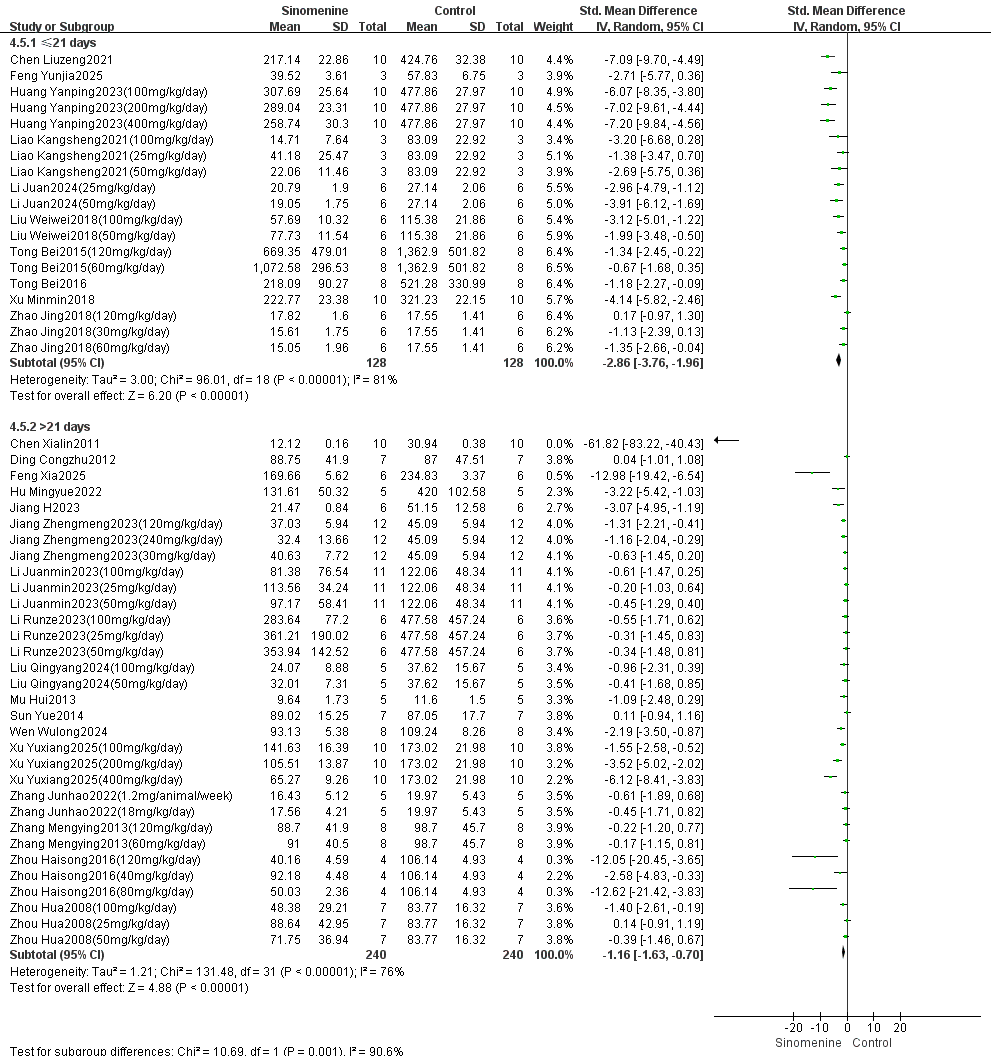
**

**Fig S15. Subgroup analysis of IL-6 by administration route.**

**
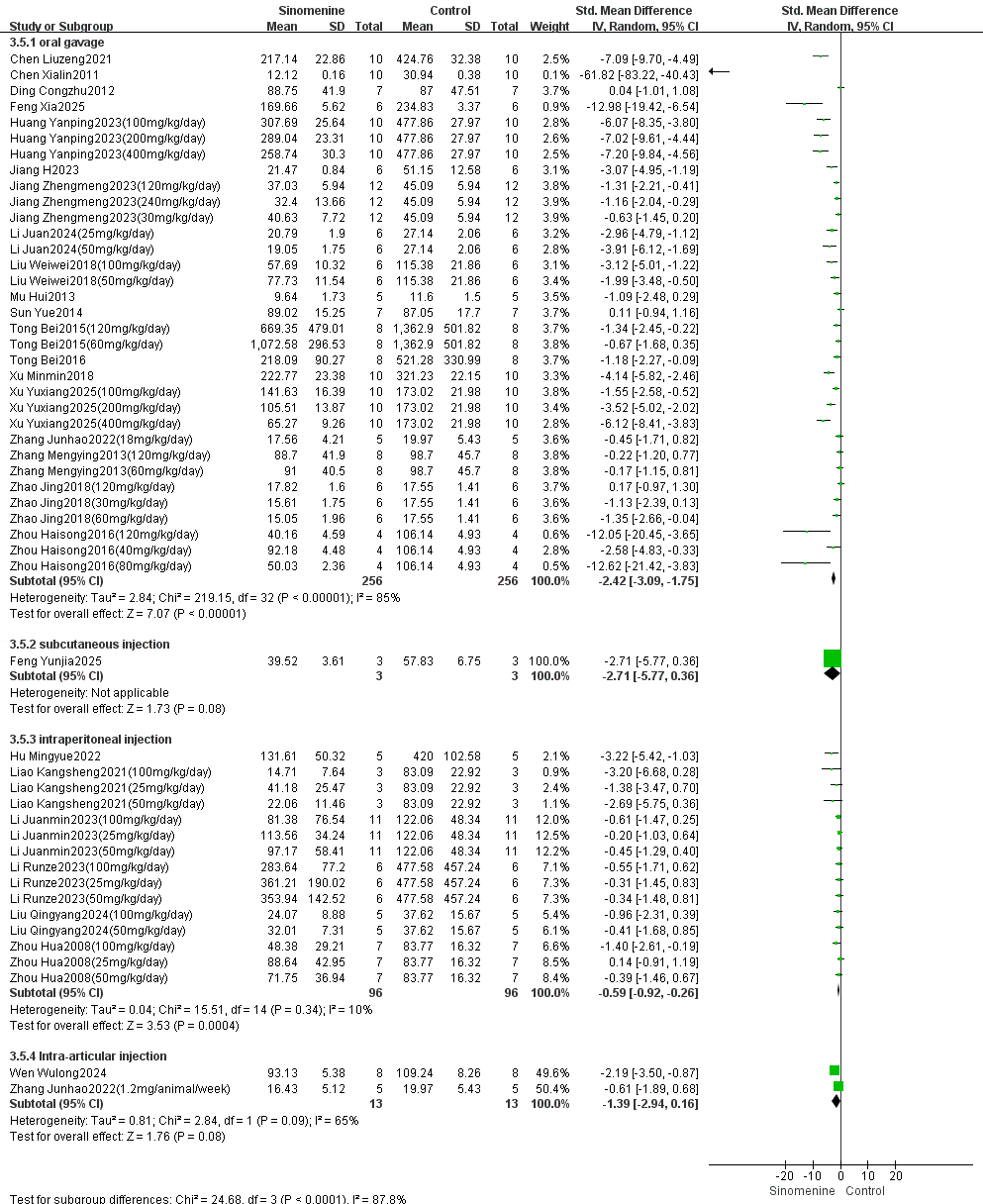
**

**Fig S16. Subgroup analysis of IL-10 by dose.**

**
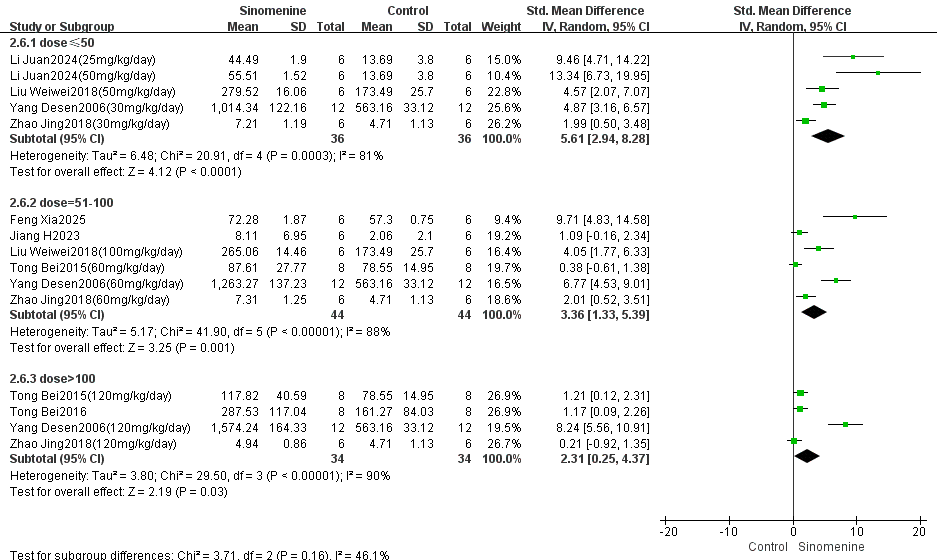
**

**Fig S17. Subgroup analysis of IL-10 by intervention duration.**

**
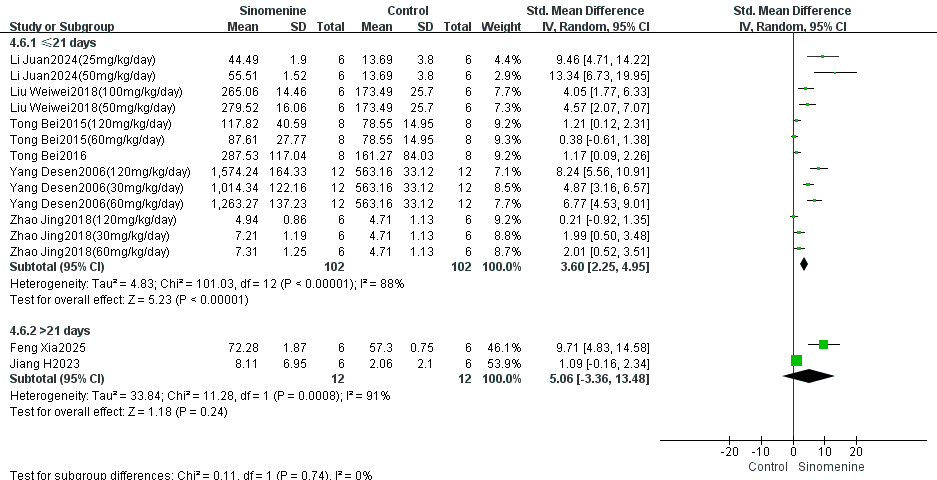
**

**Fig S18. Subgroup analysis of histological score by dose.**

**
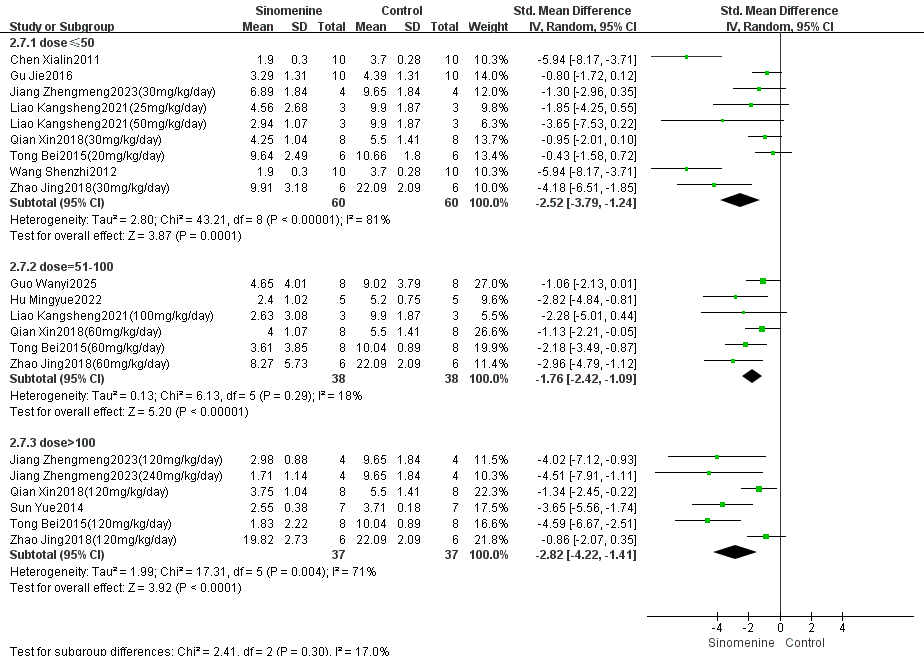
**

**Fig S19. Subgroup analysis of histological score by intervention duration.**

**
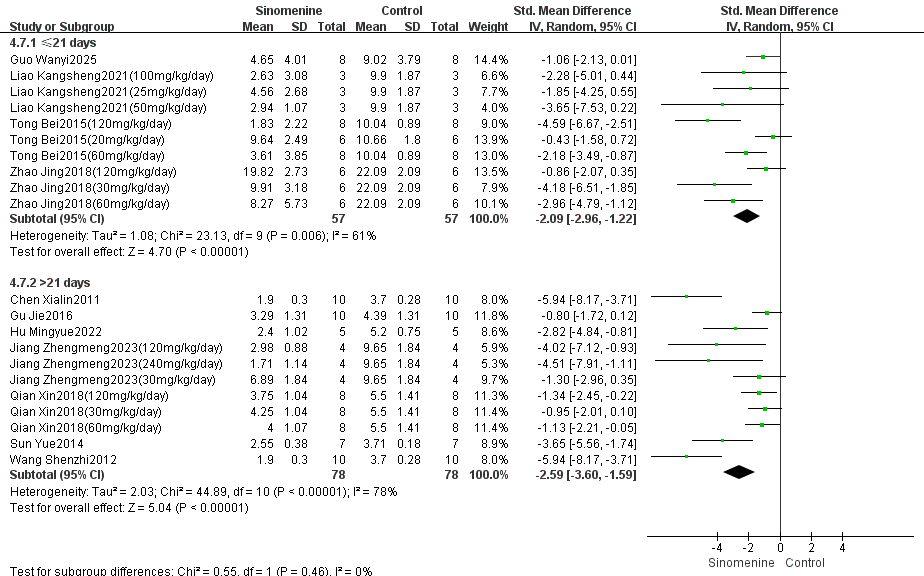
**

**Fig S20. Subgroup analysis of histological score by administration route.**

**
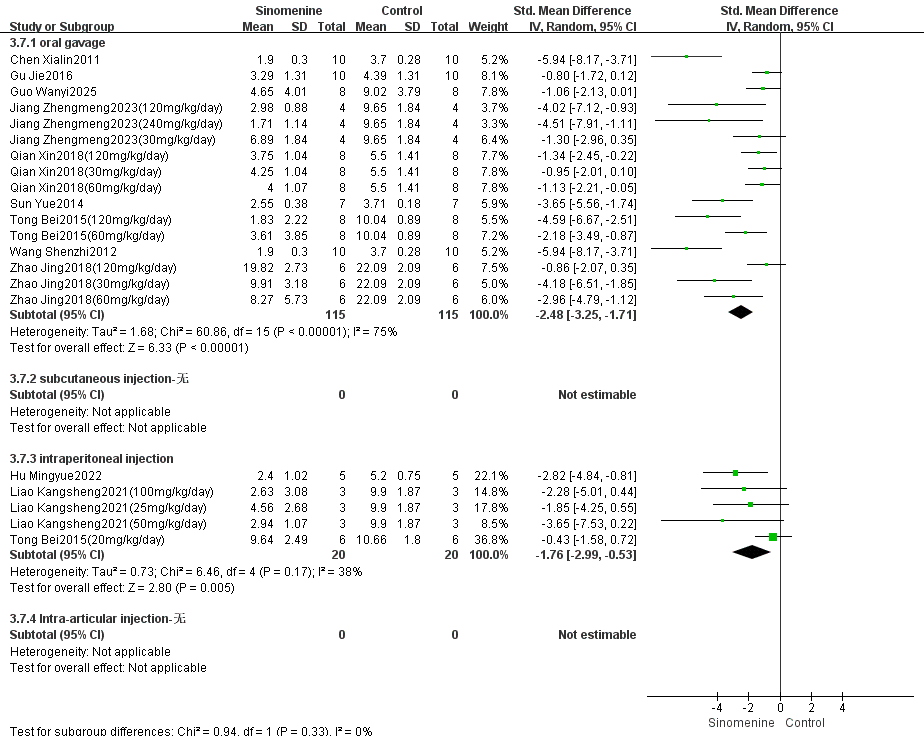
**

**Fig S21. Subgroup analysis of MMP-9 by dose.**

**
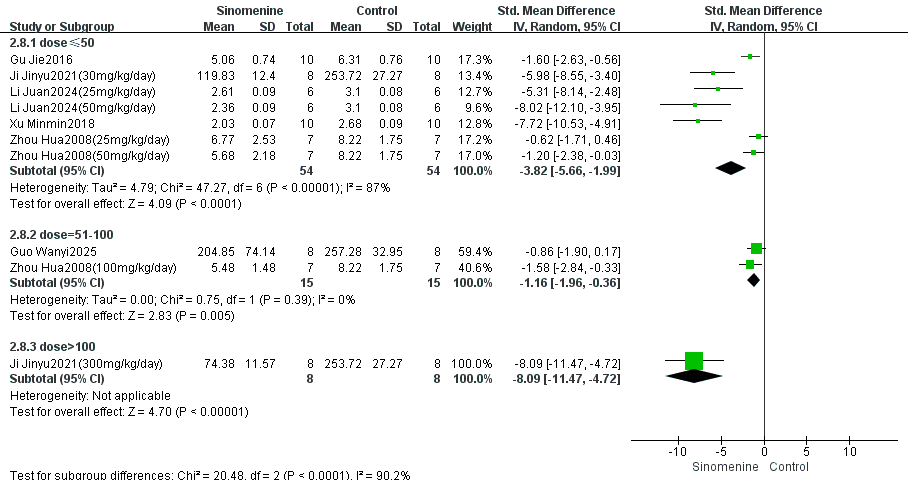
**

**Fig S22. Subgroup analysis of MMP-9 by intervention duration.**

**
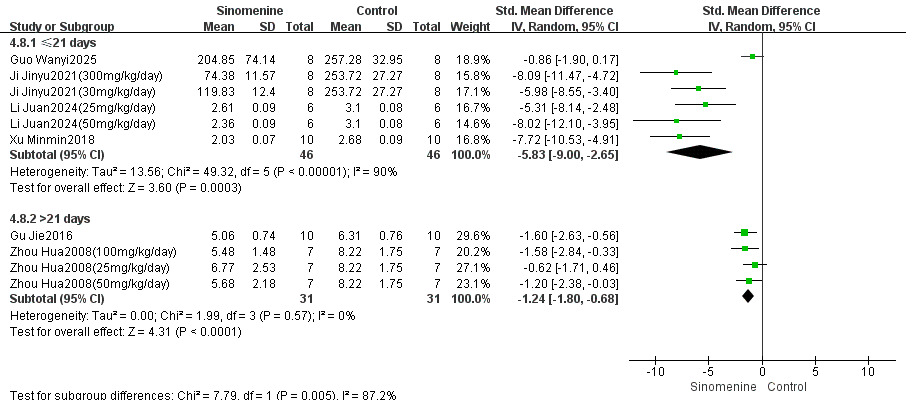
**

**Fig S23. Subgroup analysis of MMP-9 by administration route.**

**
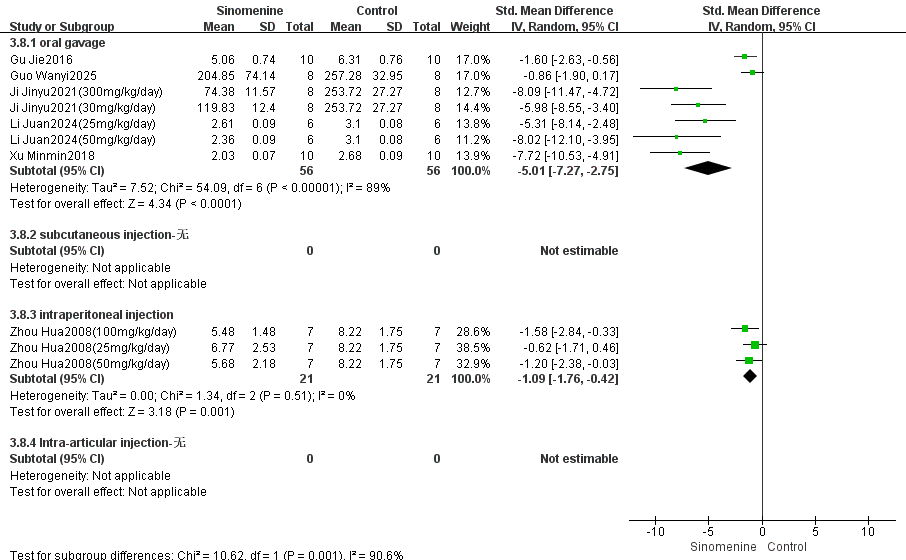
**

**Fig S24. Subgroup analysis of RANKL by dose.**

**
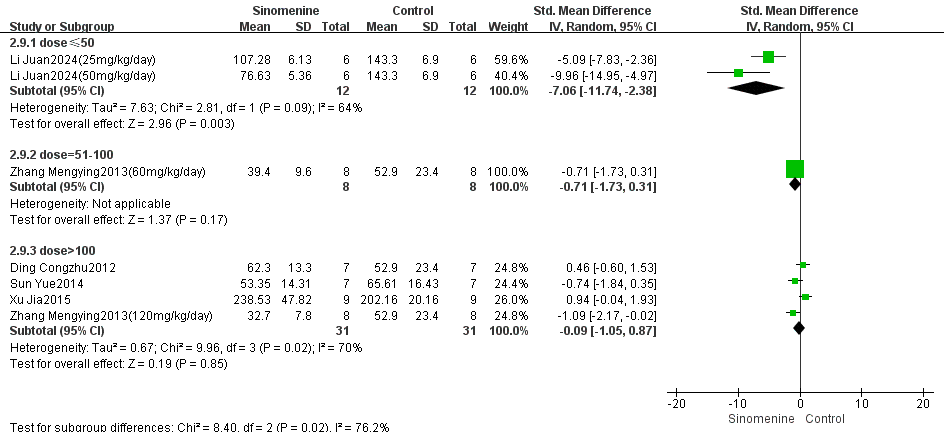
**

**Fig S25. Subgroup analysis of RANKL by intervention duration.**

**
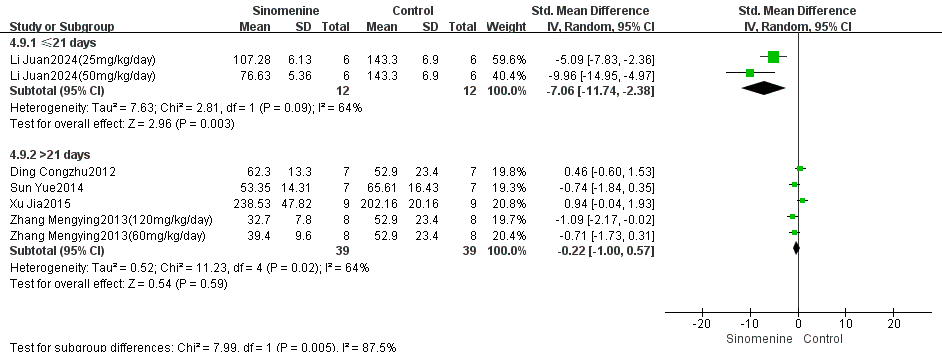
**

**Fig S26. Subgroup analysis of OPG by dose.**

**
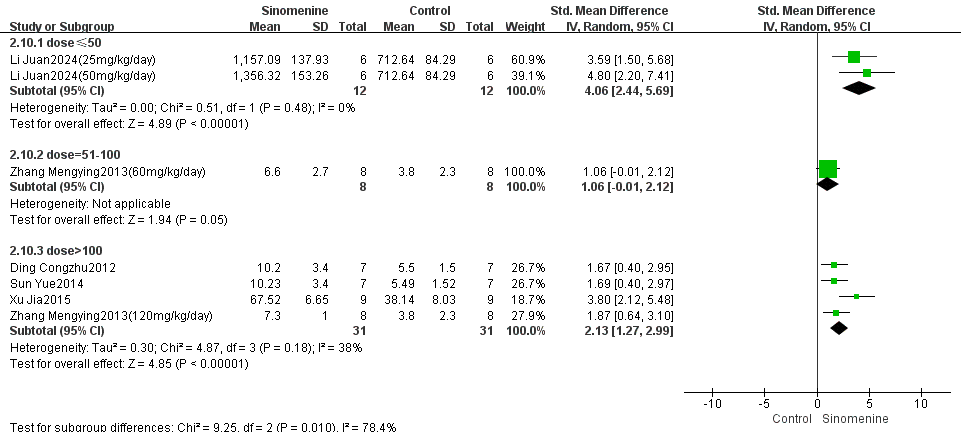
**

**Fig S27. Subgroup analysis of OPG by intervention duration.**

**
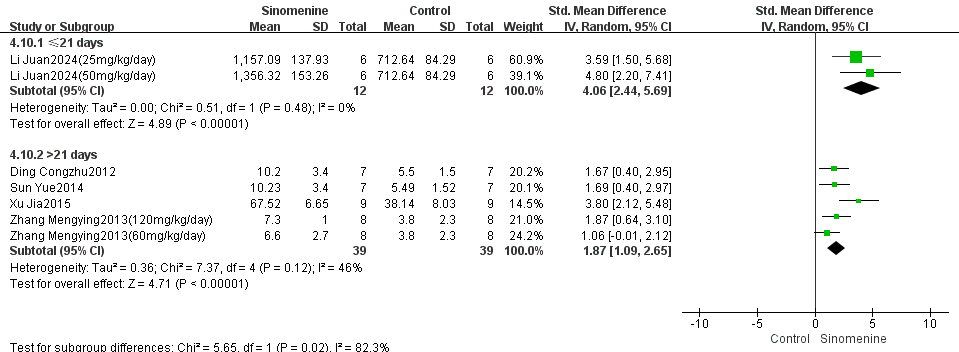
**

**Fig S28. Forest plots for ALT(A) and AST(B).**

**
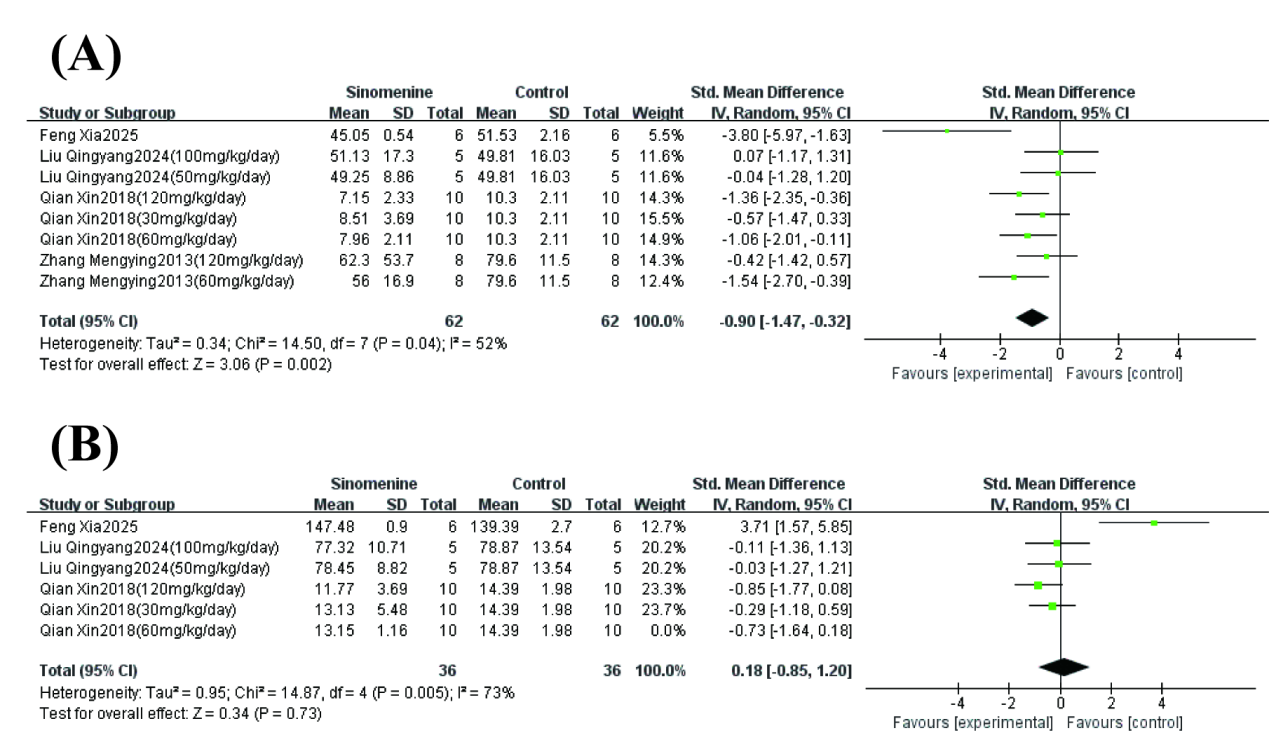
**

**Fig S29. Leave-one-out sensitivity analysis for suspect studies**

**
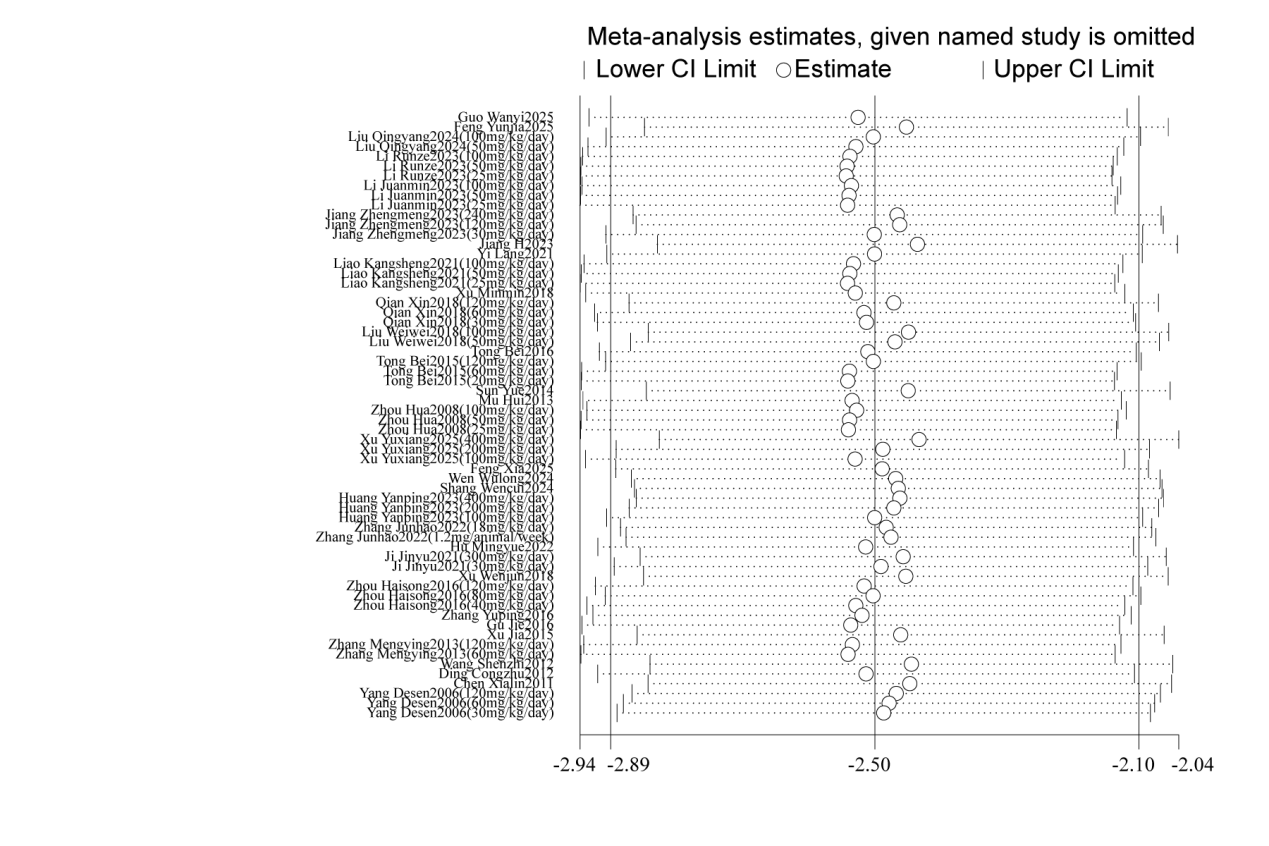
**

**Fig S30. Forest plot after excluding all seven GRIM-inconsistent studies.**

**
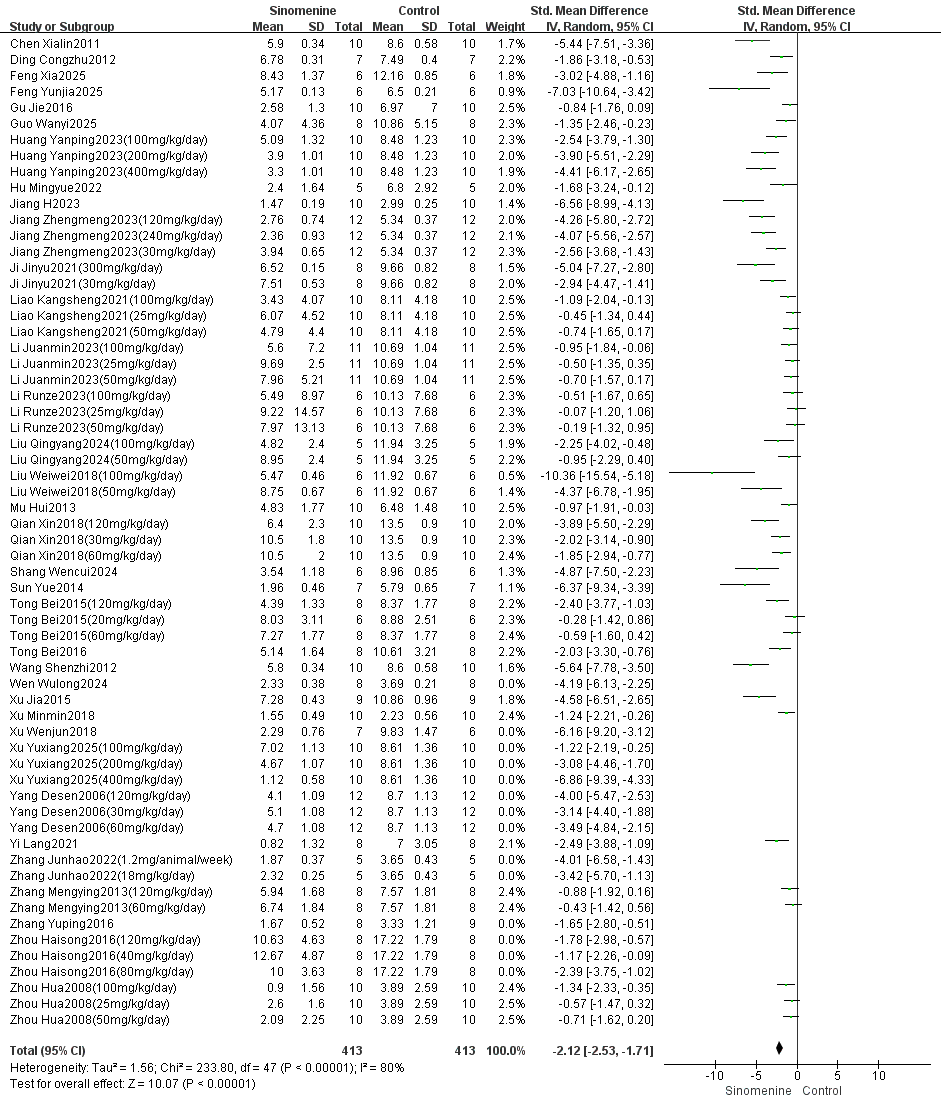
**
